# Undirected Exploration of Binding Pockets with Flexible Topology

**DOI:** 10.1021/acs.jctc.5c00825

**Published:** 2025-10-09

**Authors:** Fatemeh Fathi Niazi, Seungmin Yoon, Khadim Mbacke, Alex Dickson

**Affiliations:** † Department of Computational Mathematics, Science and Engineering, 3078 Michigan State University, East Lansing, Michigan 48824, United States; ‡ Department of Pharmacology and Toxicology, Michigan State University, East Lansing, Michigan 48824, United States; § Department of Physics and Astronomy, Michigan State University, East Lansing, Michigan 48824, United States; ∥ Department of Biochemistry and Molecular Biology, Michigan State University, East Lansing, Michigan 48824, United States

## Abstract

A common first step in drug design is virtual high-throughput screening (VHTS), where a large number of potential drug molecules are computationally modeled in a protein binding pocket and filtered down to a smaller set of hits that can be further tested computationally or experimentally. Traditional strategies for VHTS do not account for ligand-induced conformational changes in proteins as they typically rely on a single static structure to represent the protein. This neglects the role of binding entropy and the fact that different ligand molecules can induce slightly different conformations in the protein binding site that significantly affect the assessment of a given molecule’s fit. To address this challenge, we have developed a method called “flexible topology”, where a subset of atoms, typically representing a small molecule ligand, can continuously change their atomic identities, which are encoded by a set of attributes that parametrize the nonbonded interactions. These attributes are all implemented as dynamic variables that have masses and evolve over time using gradients of the energy function. In other words, the attributes feel forces from their surrounding environment and respond accordingly. In this way, by observing a set of flexible topology particles move and change in a ligand-binding site, we can learn the preferences of a binding pocket. Here, we demonstrate how undirected flexible topology simulations can be used to explore ligand-binding sites and reveal the desirable properties of potential ligands. We use the β-2-adrenergic receptor as an illustrative example and compare the properties of flexible topology particle groups with a set of 29 B2AR ligand-bound crystal structures, covering 13 distinct ligands. We also show how the shape- and electrostatics-based virtual screening software “eon” from OpenEye can be used to find hits that come as close as possible to mimicking the orientation of our flexible topology atoms.

## Graphical abstract

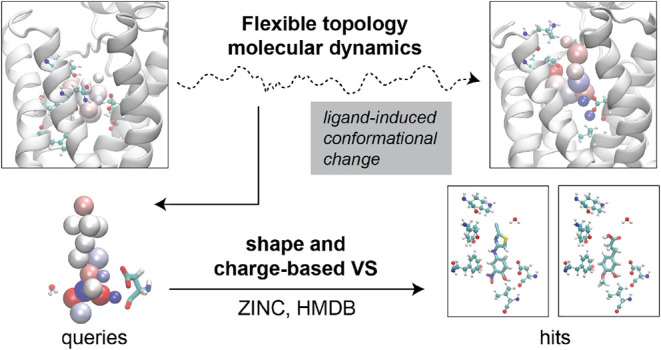

## Introduction

Proteins are naturally flexible, and their ability to adopt various conformations is fundamental to an array of biological processes.^1–5^ Ligand-protein binding often involves conformational induction, which refers to the process where a ligand induces a protein to adopt a conformation that it may not naturally take in its unbound state.^6,7^ As such, it is widely known that different ligands can induce slightly different conformations in the protein binding site.^2,7,8^

Some ligand-induced conformations involve large conformational changes, such as the glutamine-binding protein (GlnBP), which switches between an open conformation (populated >90% in the apo state) and a ligand-induced closed conformation,^9^ on time scales slower than 10 ms. Other changes are ligand-specific, as was observed in the β-1-adrenergic receptor (B1AR). Toporowska et al.^10^ used hydrogen–deuterium exchange mass spectrometry (HDX-MS) to observe that nine different ligands each induce distinct conformational changes in the receptor, particularly in the intracellular loop 1 (ICL1), which plays an important role in receptor activation and G-protein recruitment. Similarly, nuclear receptors (NR) have been shown to demonstrate specific conformational shifts in response to different ligands, which align with the ligand activities (e.g., full or partial agonism).^11^ Finally, research into cryptic binding sites—pockets in proteins that are hidden in the apo state but appears when a ligand binds—also emphasized the significance of ligand-induced conformational changes, ranging from subtle side-chain adjustments to significant structural shifts like loop movements.^12^

In addition to ligand-induced conformational changes in proteins, it has also been shown that the water molecules in the binding site are crucial for modeling protein–ligand interactions.^13,14^ For instance, ligand functional groups that replace tightly bound water molecules in the binding pocket can significantly enhance binding affinity by contributing favorable desolvation entropy.^15,16^ Water-mediated hydrogen bonds between the protein and ligand are also known to contribute significant stabilizing effects,^17,18^ which are difficult to capture or predict using continuum solvent models.

While the dynamic nature of proteins, solvation effects, and ligand-induced conformational changes play a crucial role in determining binding affinity, most existing receptor-based virtual screening (VS) techniques do not adequately account for these factors.^19^ Docking approaches typically use rigid or semiflexible receptor models, which assumes that the binding pocket remains static during ligand interaction and neglects solvation effects.^20,21^ While some flexible docking methods allow for limited side-chain flexibility,^22^ fully accounting for protein dynamics significantly increases the search space and computational cost due to the high protein degrees of freedom, making it impractical for large-scale screening.^23,24^ Among various VS methods, some have made strides to address the dynamic behavior of proteins. Ensemble docking partially addresses this by using multiple pregenerated receptor conformations, but it remains constrained by the availability of experimentally determined structures and the challenges of accurately scoring different protein conformations.^23^ Flexible docking algorithms, such as those implemented in RosettaVS, consider side-chain movements to improve docking accuracy.^6^ gEDES predicts bound-like conformations in flexible targets by using enhanced sampling methods to generate potential alternative pocket shapes and then docking in small molecules.^25^ However, these methods are still limited by either only considering limited motions or neglecting conformational changes that are induced by the presence of a ligand.

How can we examine ligand-induced conformational changes for a large set of ligands without running separate MD simulations for each one? This motivated our development of the Flexible Topology algorithm, which uses a set of particles that can fluidly change their bonding and chemical type, representing many different compounds within a continuous MD simulation. The identities of atoms are encoded by three nonbonded parameters: partial atomic charge (*q*), Lennard-Jones radius (σ), and Lennard-Jones well depth (ε). These parameters, collectively referred to as “attributes”, are all implemented as dynamic variables, which have masses and evolve in time using gradients of the energy function. In other words, the attributes feel forces from their surrounding environment and respond accordingly. In the original approach,^26^ FT simulation starts with an amorphous blob of FT particles that were guided to assemble into a target ligand using a deterministic loss function. The loss was defined as the difference between the atomic environment vectors (AVEs) of the FT atoms and those of a particular set of target atoms. The AVEs, derived from Behler-Parrinello symmetry functions,^27^ provided a translation- and rotation-invariant representation of the local environment of each atom.

Here, we introduce the “undirected” Flexible Topology Pocket Explorer (FTPE) method, where FT particles move and change within a ligand-binding site without being guided toward a predefined target molecule. In this way, we can learn the preferences of a binding pocket while capturing the ligand-induced changes in protein conformation and binding pocket solvation. In this work, we also demonstrate how undirected flexible topology simulations can be used for virtual screening by searching for compounds that best match the particle distributions. Our application system is the orthosteric binding pocket of the β-2-adrenergic receptor (B2AR), which is among the best-studied G-protein-coupled receptors (GPCR).^28,29^ B2AR sits in the cell membrane and responds to epinephrine (adrenaline) by undergoing a conformational change that activates binding of an internal G-protein trimer, ultimately leading to cAMP signaling.^30^ This can trigger a number of physiological effects, including smooth muscle relaxation and increased heart rate.^31^ B2AR is a widely studied drug target in respiratory diseases, with agonists like albuterol and salmeterol used to treat asthma and COPD by relaxing airway muscles and β-blockers such as propranolol acting as antagonists, preventing receptor activation to reduce heart rate and blood pressure.^32^ Notably, agonists and antagonists bind in the same orthosteric site, highlighting the significant role of ligand-induced conformational changes and allostery in B2AR.

To demonstrate and validate the FTPE method, we analyzed a set of 13 unique known B2AR ligands and compared them to the template generated by FT particles. We first evaluate the influence of FTPE simulation parameters by measuring the electrostatic RMSD and shape overlap between the generated frames and known ligands, which enabled us to identify optimal parameters for our simulations. We then examine how FT frames capture the spatial envelopes and electrostatic potentials of these ligands as well as the polar interactions formed with specific residues. Finally, we use shape- and electrostatic-based ligand screening tools to identify compounds from two ligand databases (ZINC^33^ and hmdb^34^) that best overlap with individual FT frames, demonstrating the potential use of this method in an early-stage drug discovery project.

## Methods

### Preparation of β-2-Adrenergic Receptor System

The B2AR system was prepared for molecular simulation using the Membrane Builder tools from CHARMM-GUI,^35^ with PDBID:4LDL^36^ as a starting point, which contains a bound ligand (hydroxybenzylisoproterenol, referred to as XQC below), an engineered nanobody on the intracellular side, and human B2AR with a chimeric N-terminal lysozyme fragment. In our structure, we omit the lysozyme fragment, including only residues 1030–1342 on chain A, which correspond to residues 30–342 in the conventional B2AR sequence. Two disulfide bonds are maintained on the extracellular side: Cys106-Cys191 and Cys184-Cys190. Both the ligand and stabilizing nanobody are omitted. Residues Glu231 and Lys263 are modeled as connected to join their two helices on the intracellular side. The OPM 2.0 server^37^ is used to insert the protein into the lipid bilayer.

We construct an asymmetrical membrane with 18 DOPC, 25 DOPE, 8 DOPS, 10 OSM, and 30 CHL on the intracellular side and 37 DOPC, 3 DOPE, 0 DOPS, 22 OSM, and 31 CHL on the extracellular side. The mixture of lipid types followed a previous study by Ingolfsson et al.^38^ while keeping the lengths of lipid tails roughly constant. It is important to note that each DOPS carries a net negative charge, and the other lipid types are neutral, creating a physiological polarization of the membrane. The system is neutralized and raised to a 0.15 M ionic strength with 25 sodium ions and 27 chloride ions. After solvating the system using a 10 Å cutoff, the system has 66839 atoms and a periodic box size of (*L*_*x*_, *L*_*y*_, *L*_*z*_) = (81.4, 81.4, 112.9)­Å. The system is equilibrated by using the standard protocol from the CHARMM-GUI Membrane Builder. All minimization and subsequent dynamics are run in OpenMM v8.1.1.^39^

### Initialization of Flexible Topology Particles

The conformation at the end of the equilibration trajectory is used to initialize the flexible topology simulations. The binding citric centroid is determined as the center of geometry of the following residues: Trp109, Thr110, Asp113, Val117, Cys191, Asp192, Phe193, Ala200, Ser203, Ser207, Phe289, Phe290, Asn293, Ile309, Asn312, and Tyr316. The closest 13 waters to the centroid are identified and deleted from the structure to make room for the flexible topology (FT) particles.

Initial positions for the FT particles are determined using the gen_init_pos function in the Flexible Topology repository^40^ as follows. Trial positions are drawn from a Gaussian distribution with a mean at the binding site centroid and a width of 0.9 nm. A trial position is accepted if all the following conditions are true: (1) the particle is not closer than 2.0 Å to any atom in the system, (2) the particle is not closer than 1.5 Å to any FT particle already initialized, and (3) the distance to the closest FT particle is not greater than 2.5 Å. Criteria (1) and (2) are implemented to prevent overlaps, and criterion (3) enforces that the FT particles form a single contiguous entity. This procedure is continued until all *N* particles are placed in the pocket. If more than 10,000 trials have been attempted and the particles are still not successfully placed, the algorithm starts over again. Up to 10 restarts are allowed by default, and if the maximum number of restarts is exceeded, the program is terminated with an error message instructing the user to delete more water molecules or consider a smaller number of particles. In this work, values of *N* between 8 and 20 are used.

Initial attributes are randomly picked from a uniform distribution between the bounds shown in Table 1. The charges are restricted to be somewhat neutral upon initialization to encourage charged groups to evolve more naturally. The sigma values are restricted to be at the minimum value to help reduce overlaps.

**Table 1:** Upper and Lower Bounds for the Values of Different Attributes both during Initialization and during the Simulation^a^

	initial		during sim.	
attribute	lower bound	upper bound	lower bound	upper bound
charge	–0.01	0.01	–0.5	0.5
epsilon	0.03	1.50	0.03	1.50
sigma	0.20	0.20	0.20	0.36

a In the latter case, the bounds are enforced in the integrator.

### Custom Forces for Flexible Topology Particles

To enable the treatment of charge, epsilon, and sigma as dynamic variables, as well as to productively restrain the positions and attributes during simulation, we employ a series of custom forces.

#### Continuity Force

As previously described,^26^ we have implemented and here use “OpenMM Continuity Force”^41^ as an OpenMM plugin to ensure that all particles remain continuously connected in space. This operates by classifying particles into connected components on-the-fly and adding an attractive force between pairs of particles from components that exceed a user-defined cutoff distance. The attractive energy is harmonic: *E*(*r*_*ij*_) = *k*(*r*_*ij*_ – *d*)^2^; here, we use a cutoff distance (*d*) of 2.5 Å and a force constant (*k*) of 10,000 kj/mol/nm^2^.

#### AttrForce

An additional force is implemented that operates only on the attributes of each particle (Lennard-Jones radius (σ), partial charge (*q*), and Lennard-Jones well depth (ε)). We refer to this force as “AttrForce” and it is included in the Flexible Topology toolkit. AttrForce was developed by inspecting the values of atomic attributes for 14,001 molecules,^42^ previously built starting from the PHYSPROP data set,^43^ after parametrizing each molecule using CGenFF.^44^ Figure S1A shows a simple relationship between the values of *q* and σ, where *q* is unconstrained at higher σ, but restricted to a small range ([0.0,0.6]) for σ < 0.3. This is chemically intuitive, as the only low-σ atoms in the data set are hydrogens, which have a small partial positive charge. Similarly, there is a simple relationship between σ and ε (top right of Figure S1B), where ε is restricted to [0.0–0.5] for σ < 0.27 and is unrestricted for σ > 0.3. No such relationship was observed between ε and *q*.

These two relationships are mimicked using the following potential energy restraint, applied separately to each FT particle:

1 $$V_{\mathrm{AF}}\left(q,\sigma ,\varepsilon \right)=\mathrm{Af}\left(\sigma \right){\left(\varepsilon -0.25\right)}^{2}+\mathrm{Bf}\left(\sigma \right){\left(q-0.25\right)}^{2}$$

where *f*(σ) is a sigmoidal function:

2 $$f\left(\sigma \right)=\frac{1}{1+e^{C\left(\sigma -0.3\right)}}$$

The parameter *C* was chosen to exhibit fast switching between 1 and 0 as σ crosses 0.3. The parameters *A* and *B* were chosen to maximally improve the likelihood of the observed attributes over a set of attributes randomly selected from a normal distribution with the same mean and variance of the observed data. The values obtained using this procedure are *A* = 941.5 and *B* = 275.3. AttrForce is implemented in OpenMM as a “CustomCVForce” and is available in the Flexible Topology toolkit.

#### Centroid Bond Force

To ensure that the particles remain in the binding site of interest, we implement a CentroidBondForce object in OpenMM. This acts on each of the FT particles individually as follows:

3 $$V_{\mathrm{CBF}}\left(\mathrm{x⃗}\right)=0.5k_{\mathrm{CBF}}H\left(d_{\mathrm{COM}}\left(\mathrm{x⃗}\right)-d_{0}\right){\left(d_{\mathrm{COM}}\left(\mathrm{x⃗}\right)-d_{0}\right)}^{2}$$

where *d*_COM_(*x⃗*) is the distance from *x⃗* to the binding site centroid. The parameter *d*_0_ is set to 0.9 nm, and *k*_CBF_ = 1000 kJ/mol/nm^2^. The Heaviside step function *H*(*a*) is equal to 1 for *a* > 0 and 0 for *a* < 0. In this way, the centroid bond force is activated only when a particle is farther than *d*_0_ from the binding site centroid.

#### Nonbonded Interactions

One of the most significant aspects of our implementation is how we calculate the nonbonded interactions between the FT particles and system atoms. This requires much modification to typical nonbonded energy calculations in OpenMM, as the parameters that govern the interactions change with each time step. Each atomic attribute is defined as a public variable in OpenMM that is accessible to all forces and to the integrator. The nonbonded forces for each FT atom are implemented as a separate CustomNonbondedForce, with energy function

4 $$V_{FT-nb}\left({\mathrm{x⃗}}_{i},q_{i},\sigma_{i},\varepsilon_{i},X\right)=\sum_{j}4\varepsilon_{\mathrm{ij}}\left[{\left(\frac{\sigma_{\mathrm{ij}}}{r_{\mathrm{ij}}}\right)}^{12}-{\left(\frac{\sigma_{\mathrm{ij}}}{r_{\mathrm{ij}}}\right)}^{6}\right]+k_{e}\frac{q_{i}q_{j}}{r_{\mathrm{ij}}}$$

where σ_*ij*_ = (σ_*i*_ + σ_*j*_) /2, $\varepsilon_{ij}=\sqrt{\varepsilon_{i}\varepsilon_{j}}$ , and *r*_*ij*_ is the distance between the system atom (*j*) and the FT particle (*i*). The constant *k*_*e*_ is the Coulomb constant, 138.935456 (kJ/mol) nm *e* ^–2^, where *e* is the unit of elementary charge.

### Flexible Topology Simulations in B2AR

The positions and attributes evolve forward in time using a custom integrator (CustomHybridIntegratorRestrictedChargeVariance in the Flexible Topology toolkit). As described previously,^26^ this integrator evolves the positions with Langevin dynamics and the attributes with Brownian dynamics. This treatment avoids ascribing velocities to the attributes and simplifies the implementation of attribute bounds. The attribute bounds are specified in Table 1, and constrain the sum of the charges to a specified quantity (here, zero). It also limits the variance of the charges (*S*_*q*_ = 1/*n* ∑_*i*_ (*q*_*i*_ *q̅*)^2^), by dividing each charge by $\sqrt{S_{q}/S_{\max}}$ when *S*_*q*_ > *S*_max_. We determined an appropriate value for *S*_*q*_ by examining the root-mean-squared partial charges of the set of B2AR-binding ligands. The average root-mean-squared partial charge was 0.269*e* with a standard deviation of 0.039 across ligands. We found that an *S*_*q*_ value of 0.08*e* ^2^ is appropriate, resulting in values of root-mean-squared partial charge that tightly range between 0.280 and 0.283.

After initialization and addition of custom forces to the system, the attributes are energy minimized, allowing particle sizes to increase and the charges to deviate from neutral, where it is energetically favorable. The positions of the entire system are briefly minimized by using the standard OpenMM energy minimization function, with a maximum of 1000 steps and a tolerance of 100 kJ/mol. A step size of 2 fs is used for all dynamics reported here. The system is run for 100,000 steps (200 ps) at *T* = 10 K with Harmonic restraints of 400.0 kcal/mol/nm^2^ on the protein backbone and 40.0 kcal/mol/nm^2^ on the remainder of the protein. The restraints are removed for the remainder of the simulation, which is conducted for 100,000 steps at each of the temperatures 10, 20, 50, 100, 150, 200, 250, and 300. At the production temperature, we collect 10^6^ steps (2.0 ns) of data, saving every 2000th frame. Below we report simulations with production temperatures of 200, 250, and 300 K. For temperatures below 300 K, we truncate the heating process but still collect 2.0 ns of data at the production temperature.

### Alignment and Analysis of Known B2AR Binders

A set of 33 ligand-bound B2AR structures were collected from the Protein Data Bank. Following sequence alignment, a consensus binding pocket sequence was constructed. Structures that showed alterations in the binding pocket residues (3kj6, 3sn6, 6ps0), had covalently bound ligands (4qkx), or did not have ligands sufficiently overlapping the binding pocket (3pds) were discarded. Only small molecule ligands in the binding pocket were kept, which are summarized in Table 2.

**Table 2:** Ligand-Bound Structures for B2AR^a^

PDB ID	Chain IDs	ligand name
2rh1	0	CAU
3d4s	0	TIM
3ny8	0	JRZ
3ny9	0	JSZ
3nya	0	JTZ
3p0g	0	P0G
4gbr	0	CAU
4lde	0	P0G
4ldl	0	XQC
4ldo	0	ALE
5d5a	0	CAU
5d5b	0	CAU
5d6l	0	CAU
5jqh	0,1	CAU
5x7d	0	CAU
6e67	0,1	P0G
6mxt	0	K5Y
6n48	0	P0G
6oba	0	JTZ
6prz	0	JTZ
6ps1	0	TIM
6ps2	0	JTZ
6ps3	0	CVD
6ps4	0	JRZ
6ps5	0	SNP
6ps6	0	TIM
7xk9	0	GJ6
7xka	0	G1I

a For PDB structures with multiple B2AR chains specified in the “Chain IDs” column, each chain is treated as a separate system.

To facilitate further analysis of the ligands in the B2AR binding site, we predicted the protonation states of the protein and ligand, added missing residues, replaced nonstandard residues, and added solvent using PDBFixer.^45^ The OpenFF Toolkit^46^ was used to parametrize the small molecule ligands and build an OpenMM-compatible topology. OpenMM v8.1.1 ^39^ was then used to run an energy minimization for each system, followed by 10,000 steps of dynamics at 300 K. This entire procedure was simply meant to allow for the relaxation of hydrogen positions in the ligand and protein binding site and provide a more realistic description of the local electrostatic environment. Finally, one system (2rh1) was assigned as the reference, and each system was aligned to it using the backbone atoms of homologous residues in the binding pocket. For this purpose, binding pockets were defined as the set of residues that had at least one atom within 5.0 Å of an atom in the ligand.

### Electrostatic Similarity Measurements

To assess how accurately our approach can reproduce the electrostatic environment of real molecules and further, to evaluate its applicability in virtual screening, we performed electrostatic similarity analyses using EON in OpenEye. A given frame from flexible topology is converted to the mol2 format, containing artificial atom types (here, Carbon) and the partial charges of each atom. Two databases are used in this work. First, a total of 1.2 million compounds were randomly selected from the “lead-like” subset of the ZINC database (∼383 million compounds),^33^ which applies physicochemical filters suitable for early-stage drug discovery. We also screen against the Human Metabolome Database,^34^ which contains ≈217,920 small molecule metabolites found in the human body. Entries in the databases are preprocessed with OpenBabel^47^ to add hydrogens and compute partial charges for each atom using the MMFF94 method.^48^ The EON program is run with the −charges existing option, to avoid extraneous processing of the flexible topology frames (e.g., adding hydrogens). The Shape Tanimoto and Charge Tanimoto scores are collected for analysis, as well as the aligned structure of the database molecules to the flexible topology query. For EON to compute overlaps for a single frame against 1.2 million structures took roughly 1 h on a single Intel Xeon processor.

## Results

### Analysis of Known B2AR Binders

Of the 28 PDB structures with ligands in the transmembrane binding pocket, there are 13 unique ligands, as categorized in Table 3. These have molecular weights ranging from 183.204 for the endogenous agonist adrenaline to 415.5656 for the partial agonist salmeterol. Two-dimensional (2D) structures of the ligands are shown in Figure 1. Unsurprisingly, many of the ligands contain substructures based on the 3,4-dihydroxyphenyl group of adrenaline, such as G1I, GJ6, and XQC (highlighted in green). Nearly all of the ligands mimic the chiral hydroxyl group and neighboring NH–CH3 groups (highlighted in orange). As the compounds G1I and GJ6 are very similar, for the purposes of computing similarities across structures, GJ6 is omitted from the set.

**Table 3:** Ligands Found in B2AR Crystal Structures Sorted by Their Molecular Weight

ligand	ligand name	activity	mol. wt.	SMILES
ALE	adrenaline	agonist	183.204	c1(cc(c(cc1)O)O)[C@@H](O)CNC
G1I	*c*-Epi	agonist	209.242	c1cc(c(c2c1[C@H]([C@@H](CC2)NC)O)O)O
GJ6	(*R*,*R*)-*c*-ISO	agonist	237.295	c1(c2c([C@H]([C@@H](CC2)NC(C)C)O)ccc1O)O
JTZ	alprenolol	antagonist	249.349	CC(NC[C@H](O)COc1ccccc1CC=C)C
SNP	S-propanol	inverse agonist	259.343	c1(cccc2ccccc12)OC[C@@H](O)CNC(C)C
JRZ	ICI 118151	inverse agonist	277.402	C1CCc2c1c(C)ccc2OC[C@@H](O)[C@@H](NC(C)C)C
CAU	carazolol	partial inverse agonist	298.379	O[C@@H](CNC(C)C)COc1cccc2c1-c1c(N2)cccc1
TIM	timolol	partial inverse agonist	316.42	C1COCCN1C1=NSN=C1OC[C@@H](O)CNC(C)(C)C
XQC	HBI	agonist	317.38	CC(C)(Cc1ccc(cc1)O)NC[C@@H](c1ccc(c(c1)O)O)O
JSZ	compound 2	inverse agonist	335.395	C(=O)(OCC)C1=C(C)c2c(O1)cccc2OC[C@@H](O)CNC(C)C
P0G	BI-167107	agonist	370.442	Cc1ccccc1CC(C)(C)NC[C@H](O)c1ccc(O)c2NC(=O)COc12
CVD	carvedilol	agonist	406.474	O[C@H](COc1cccc2c1-c1ccccc1N2)CNCCOc1ccccc1OC
K5Y	salmeterol	partial agonist	415.5656	OCC1=C(O)C=CC(=C1)C(O)CNCCCCCCOCCCCC1=CC=CC=C1

**Figure 1:**
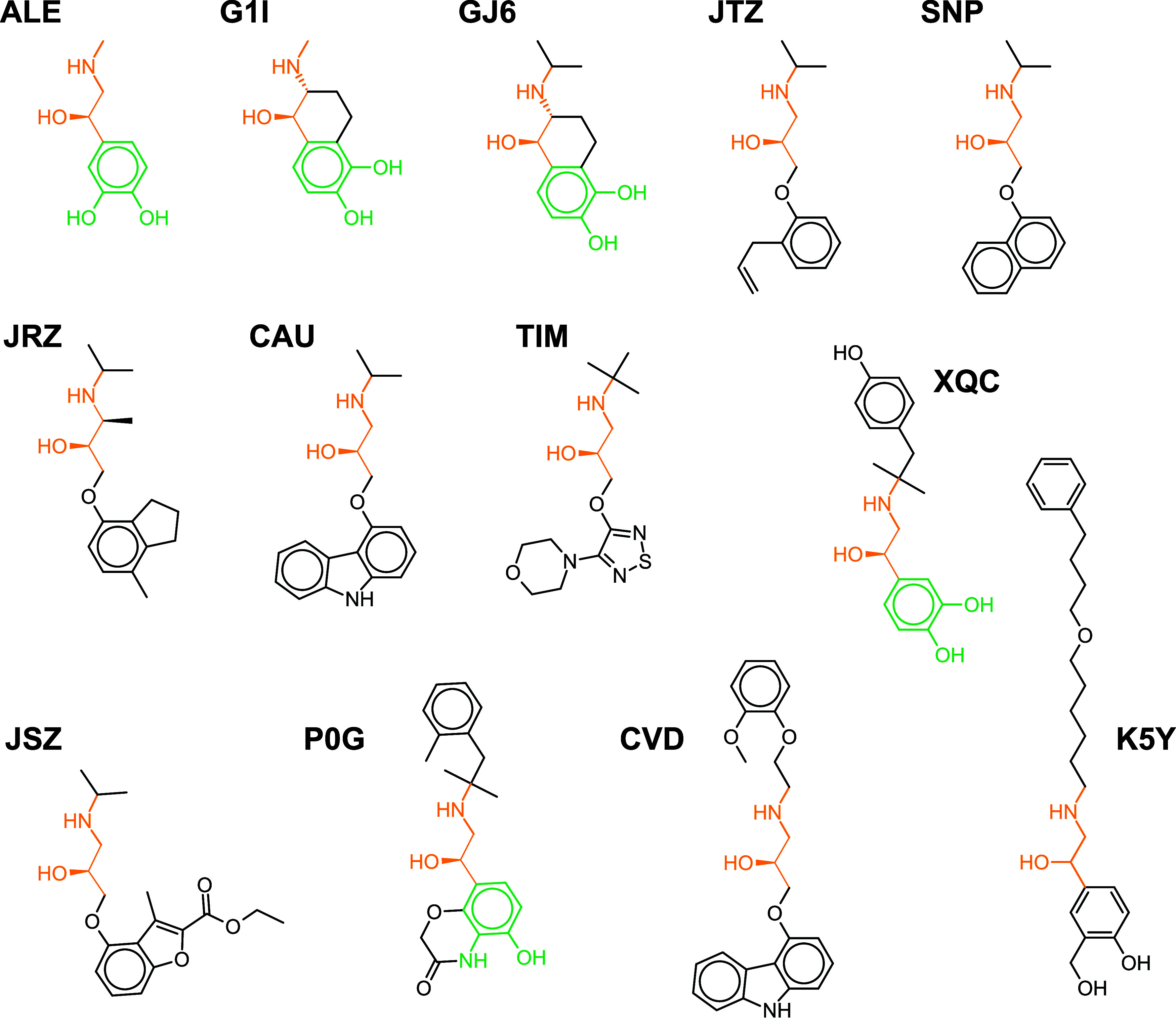
2D structures of all unique ligands in the B2AR PDB set. Ligands are arranged by ascending molecular weights, as in Table 3. Common substructures shared between ligands are shown in orange and green.

The aligned structures of the B2AR protein chains are shown in Figure 2A. Although there is considerable structural variation in grafted extracellular fragments (top) and G-protein fragments (bottom), the transmembrane regions and ligand-binding sites overlap well, with little variation between structures. Visualization of the aligned ligands in the binding site (right inset) shows that the two hydrogen bond donors in the C­(OH)–C–NH substructure are found to consistently interact with Asp113. To further examine these ligands, we add hydrogen atoms and perform a short MD-relaxation step for each ligand system. The positions of the ligand atoms, along with their partial charges and VDW diameters (σ) are then used to determine gridded electrostatic potentials (EPs): *V*_EP_(*x⃗*) = ∑_*i*_ *kq*_*i*_/*r*_*ix*_, where *q*_*i*_ is the partial charge of the *i*th ligand atom and *r*_*ix*_ is the distance from that atom to the grid point *x⃗*. Points inside the ligand atoms (determined by *r*_*ix*_ < σ/2) were assigned an electrostatic potential of zero to avoid singular values, as well as to account for the fact that these points are irrelevant as they are inaccessible to binding site residues. Positive and negative contours of the average ligand-induced electrostatic potential are shown as blue and red surfaces in Figure 2B. It shows a clear bifurcation with a positive region roughly extending from Asp113 to Ser207 and a complementary negative region overlapping with Asn293 and extending to the space between TM5 and TM6.

**Figure 2:**
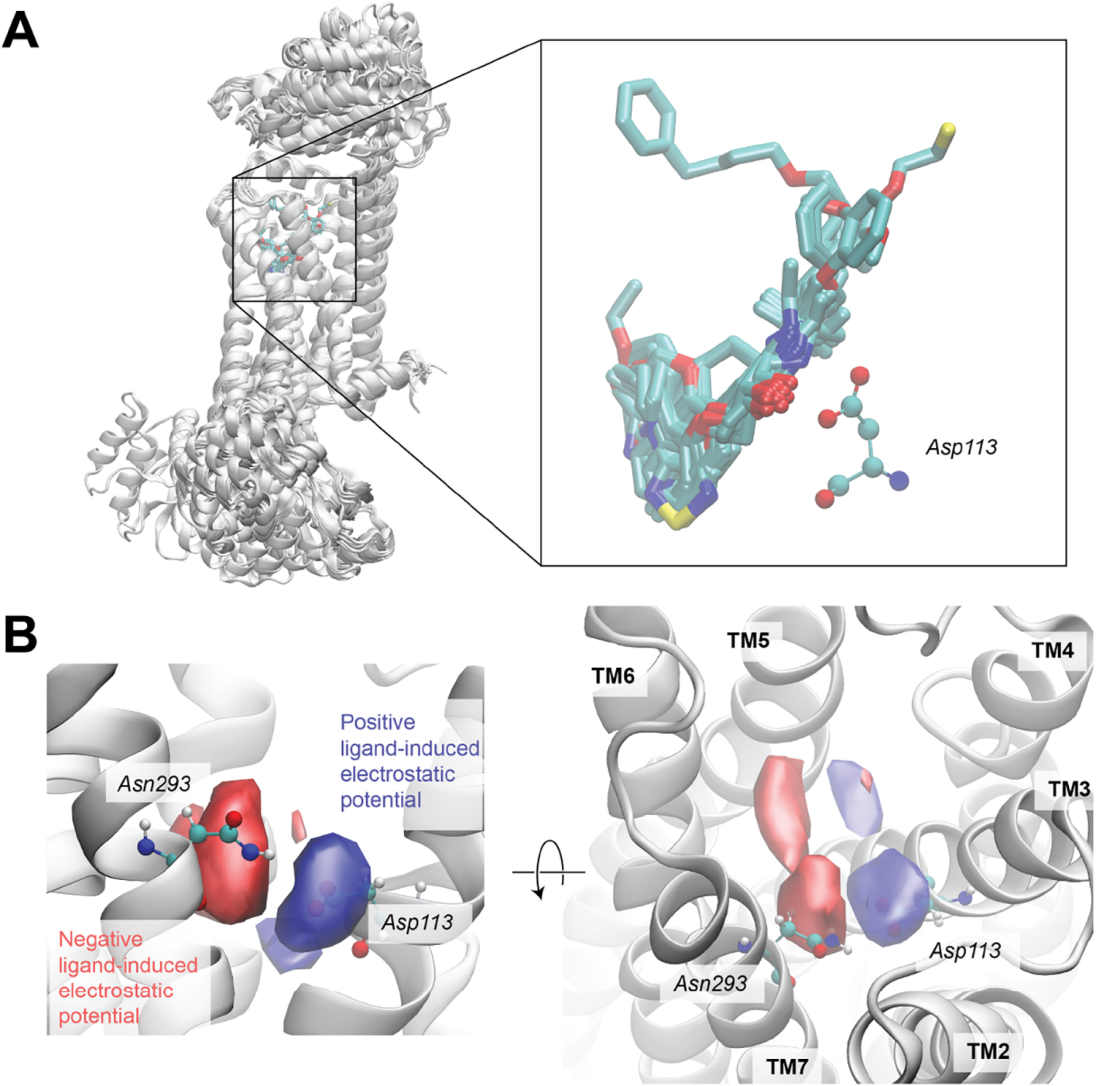
Visualization and analysis of the B2AR ligands. (A) Alignment of ligand-containing B2AR structures using the binding site residues. Individual ligands are shown in licorice representation and coloring by element (cyan = C, red = O, blue = N, yellow = S). The right panel shows a detailed overlay of the ligand poses, including their interaction with Asp113. (B) Side view (left) and top view (right) of contours of the average ligand-induced electrostatic potential in the binding site. Positive (blue) and negative (red) regions are shown using isovalues of 0.3*k* and −0.3*k*, respectively, where *k* is the Coulomb constant. Individual residues are shown that overlap with the peak regions of the electrostatic potential. Note that TM1 was removed in the side view panel for the sake of clarity.

Electrostatic potentials (EPs) for each ligand are shown individually in Figure 3, and top views are shown in Figure S2. Many of the ligands show similar EPs to the average, with a red cloud of negative density on the left and a blue cloud on the right that engulfs the negatively charged Asp113 side chain. The substituents reaching upward are either hydrophobic and not contributing to the EP (JTZ, SNP, JRZ, CAU, TIM, and JSZ) or longer and include an aromatic substituent with mixed contributions to the EP (XQC, P0G, and K5Y). Notable deviations from the average include CVD, in which there is stronger polarization and a positively charged EP region that extends upward, and in G1I, in which the red negative region is shifted further backward, away from the Asn312 side chain.

**Figure 3:**
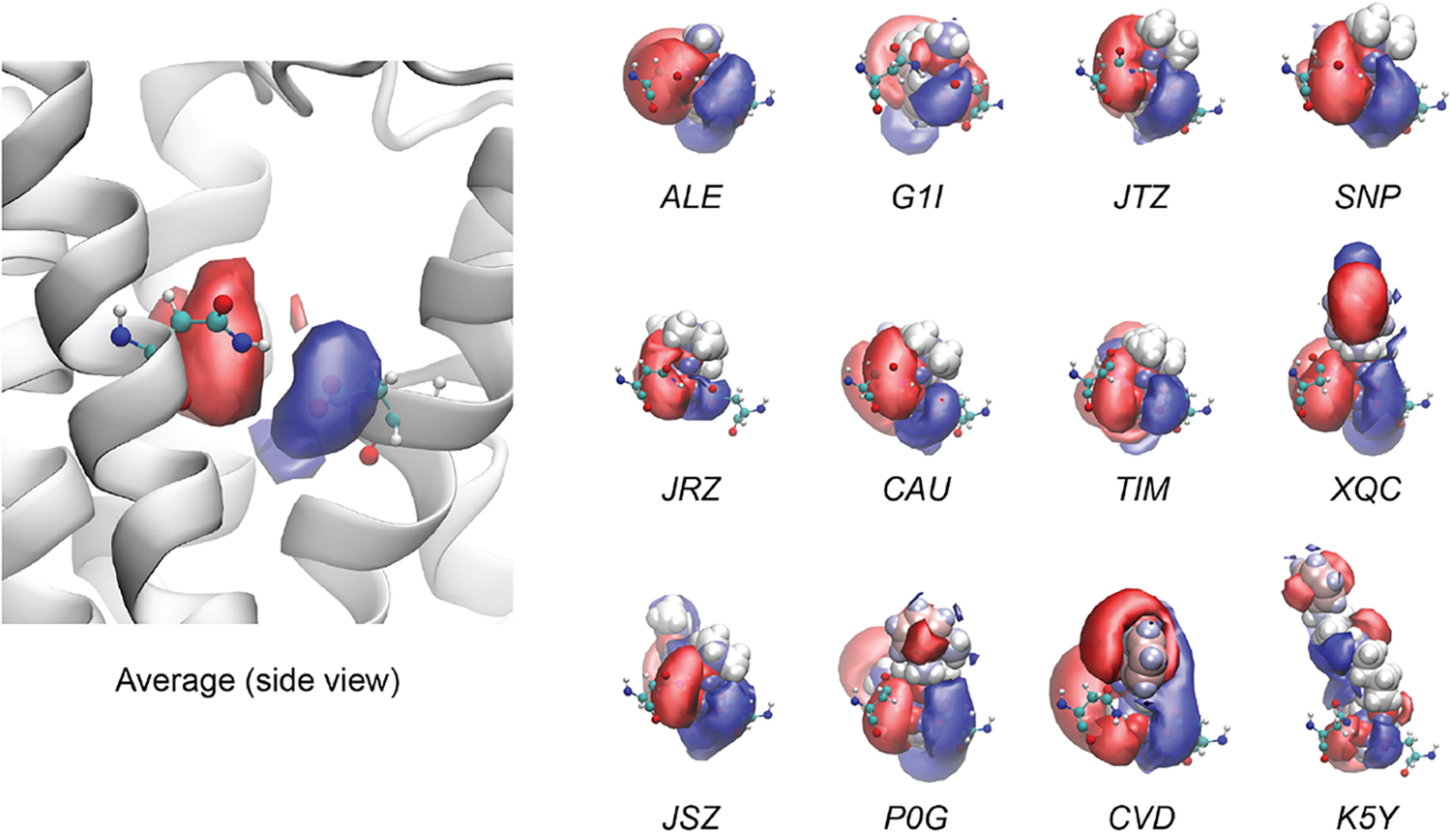
Electrostatic potential surfaces for each individual ligand. Positive (blue) and negative (red) regions are shown using isovalues of 0.3*k* and −0.3*k*, respectively, where *k* is the Coulomb constant. Ligands are shown in size-charge representation, along with two B2AR binding pocket residues in CPK representation (Asp113 [right] and Asn312 [left]). The snapshots of the average and the individual ligands were taken by using the same camera orientation after alignment of binding pocket residues.

To help calibrate the flexible topology simulations, we compute a number of ligand properties, including volume, polar surface area, and globularity. These are all computed using a grid-based representation, with a lattice size of δ = 1.0 Å. Similar to the electrostatic potential calculation, a grid point was determined to be occupied by a particle if the distance from the point to the particle was less than σ/2. The volume is then computed as the number of occupied grid cells multiplied by δ^3^. The polar surface area (PSA) is computed as the number of unoccupied cells that are adjacent to a polar cell (defined as a cell occupied by an atom with charge |*q*_*i*_|>0.3) multiplied by δ^2^. The globularity is computed as the ratio of the ligand volume to that of the minimum encompassing sphere, calculated by using a diameter equal to the largest particle–particle distance. Functions for calculating all three quantities are given in the analysis module of the Flexible Topology repository. The mean volume calculated in this manner was 355 with a standard deviation of 85 Å^3^. The minimum and maximum values were in accord with the molecular weights; the smallest ligand was ALE (211 Å^3^) and the largest was K5Y (505 Å^3^). PSA values ranged from 56 Å^2^ for JRZ to 246 Å^2^ for TIM. The average and standard deviation for the PSA were 128 ± 60. Globularity factors had an average of 0.16, ranging from 0.08 for K5Y to 0.21 for JSZ.

### Calibration of Flexible Topology Simulations in the B2AR Pocket

With the above analysis in mind, we set out to determine the extent to which sets of flexible topology (FT) particles can mimic realistic ligands in the binding pocket. In each simulation, the FT particles are initialized and then gradually brought up to the production temperature through a succession of heating steps. Frames from a representative heating trajectory are shown in Figure 4. As seen in the postminimization structure, the charges of the FT particles are initially close to zero, as can be seen from their white color. The charges then emerge quickly during the heating trajectory with the strongest positive charges forming near Asp113. These charges also spontaneously reduce their radii to the minimum allowable value, which is consistent with a hydrogen atom, in order to form interactions with the charged oxygen atoms of Asp113 as strongly as possible.

**Figure 4:**
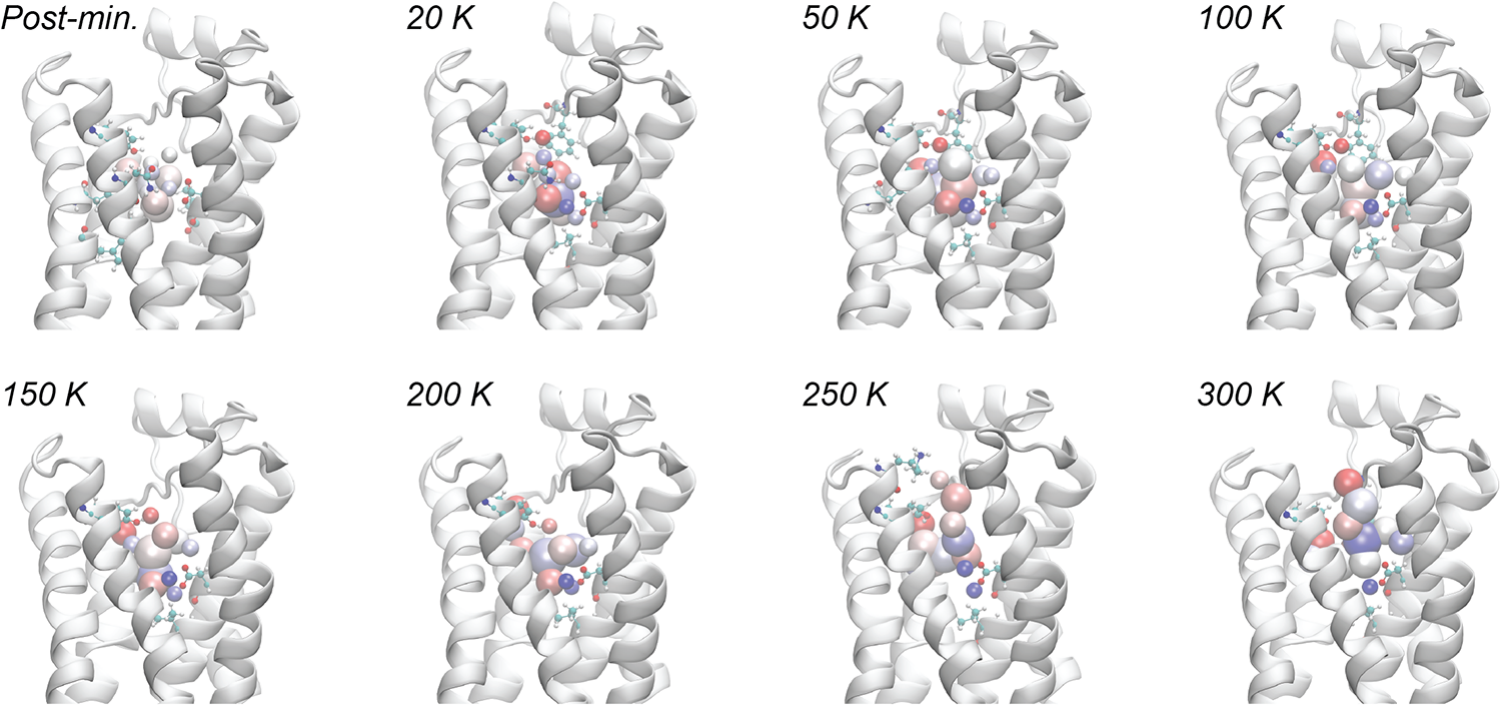
Snapshots of a flexible topology simulation during the heating phase. In each frame, a selection of B2AR residues that are within 2.5 Å of an FT particle is shown. Blue indicates a positive charge, red indicates a negative charge, and white indicates a neutral charge. Residues 1 to 50 are omitted for a clear view into the B2AR binding site.

The FT algorithm has a number of different parameters and options that can be tuned to maximize the overlap with a desired set of ligands. We first explore the impact of the number of FT particles (*N*). Obviously, larger numbers of particles will result in larger average ligand volumes, and this is observed in Figure 5A. The set of known ligands is shown in the left-most column, and the range and mean of ligand volumes sampled by a set of 5 FT simulations is shown, for different values of *N*. We find that despite the ability of the FT particles to grow in size, a single value of *N* is unable to cover the complete set of known ligands. Values of *N* = 12, *N* = 15, and *N* = 20 are chosen for further investigation, as they cover the vast majority of known B2AR ligands.

**Figure 5:**
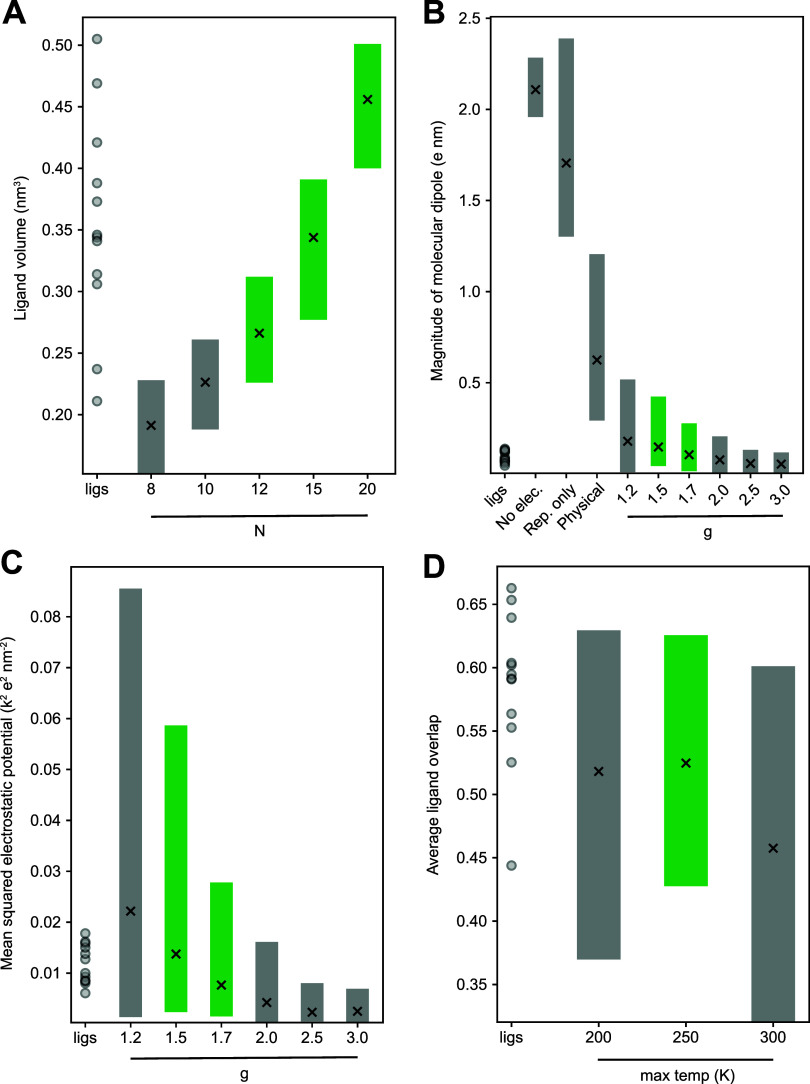
Calibration of the Flexible Topology simulations. Each panel shows the range of values of a given observable over a set of FT simulations with the specified parameters: the ligand volume as a function of the number of particles (A), the magnitude of the molecular dipole as a function of *g* (B), the mean-squared electrostatic potential as a function of *g* (C), and the average ligand overlap as a function of the maximum temperature (D). In each panel, distributions are directly compared to the corresponding values for known ligands, shown in the left-most column. Each gray bar shows the range of observed values across the set of five runs, and the “*x*” symbol denotes the mean value. The green bars in each panel show values of the parameters that were chosen for the production set of simulations. The results in (A) used *g* = 1.7 and *T* = 300 K. (B) and (D) used *N* = 15 and *T* = 300 K.

A significant question also arises in deciding how to treat electrostatic interactions between FT particles, as these are meant to mimic a set of covalently bound atoms that share electron density. One idea is to simply exclude FT-FT electrostatic interactions entirely (“No elec.” in Figure 5B). Unfortunately, under these conditions, we find that similarly charged particles tend to cluster together, creating large molecular dipoles. Another strategy is to model FT-FT electrostatic interactions but only the repulsive component (“Rep. only”). We find that this results in some moderate reduction of the dipole moment. Physical modeling of FT-FT interactions (“Physical”), in which opposite charge attraction is introduced, further helps to reduce this dipole but still results in molecules that are more polar than the set of B2AR ligands. We have found that an effective strategy is to “supercharge” the electrostatic interactions between FT particles, by introducing a constant *g* to the Coulomb equation used to calculate the electrostatic energies of FT-FT interactions

5 $$V_{\mathrm{elec}‐\mathrm{FT}}=g\sum_{i,j}\frac{kq_{i}q_{j}}{r_{\mathrm{ij}}}$$

The use of *g* factors greater than 1.0 is very effective at reducing the dipole moment of the FT particles to values that are consistent with known B2AR-binding ligands. Physically, this is promoting the juxtaposition of oppositely charged FT particles, which also occurs naturally due to polarization across covalent bonds. We further calibrated the optimal *g* value by examining the mean-squared electrostatic potential. As shown in Figure 5C, the mean value of this quantity decays with increasing *g*, and for *g* ≥ 2.0, the mean value is below the set of known ligands. For this reason, we choose values of *g* = 1.5 and *g* = 1.7 for further investigation in our production simulations.

Finally, we examine the impact of the final temperature on the average overlap of the FT simulations with known ligands (Figure 5D). While physiological temperatures are ideal for sampling relevant protein motions, we hypothesized that lower temperatures could better emphasize the most stable low-energy structures and improve agreement with the set of crystal structures of known ligands. Using *N* = 15 and *g* = 1.5, we measure the average grid-based overlap of each of the FT frames with each of the target ligands for final temperatures of *T* = 200, 250, and 300 K. We find that lower temperatures improve the average overlaps compared to 300 K, and we choose 250 K for the production simulations. Altogether, we collect six sets of production simulations, with 24–28 runs each, as shown at the bottom of Table 4.

**Table 4:** Performance of Each Run in Finding the Best Possible Matches with the Set of Known Ligands^a^

set details				shape overlap (*O*_S_)					Elec. overlap (*O*_E_)				
*N*	*g*	*T*_max_	*N*_runs_	min	avg	max	>0.6	>0.7	min	avg	max	>0.3	>0.5
8	1.7	300	5	0.45	0.60	**0.79**	5	2	**0.29**	0.39	0.52	**11**	1
10	1.7	300	5	0.49	0.64	0.74	11	2	0.24	0.35	0.44	10	0
12	1.7	300	5	0.50	0.66	0.75	10	4	0.22	0.39	0.53	**11**	**2**
15	1.7	300	5	0.58	0.66	0.71	11	2	0.21	0.34	0.46	9	0
20	1.7	300	3	0.55	0.62	0.72	8	1	0.16	0.27	0.34	4	0
15	0.0	300	5	0.28	0.37	0.47	0	0	0.00	0.01	0.04	0	0
15	R.O.	300	5	0.55	0.60	0.66	6	0	0.01	0.03	0.06	0	0
15	0.8	300	5	0.56	0.64	0.70	10	0	0.01	0.08	0.14	0	0
15	1.0	300	5	0.55	0.65	0.72	11	3	0.08	0.23	0.33	3	0
15	1.2	300	5	0.58	0.67	**0.79**	11	2	0.19	0.31	0.41	8	0
15	1.5	300	5	0.55	0.67	0.75	11	3	0.20	0.39	0.54	10	**2**
15	2.0	300	5	0.53	0.64	0.70	9	2	0.20	0.34	0.42	10	0
15	2.5	300	5	0.54	0.67	0.76	11	2	0.18	0.29	0.35	7	0
15	3.0	300	5	0.54	0.63	0.69	9	0	0.16	0.32	0.44	8	0
15	1.5	200	7	0.56	0.69	0.74	11	6	0.14	0.31	0.41	7	0
15	1.5	250	7	0.58	0.68	0.76	11	5	0.27	0.37	0.49	10	0
12	1.5	250	28	0.57	0.69	0.74	11	7	0.23	0.38	0.49	**11**	0
12	1.7	250	26	0.55	0.69	0.76	11	**8**	0.23	**0.41**	0.51	**11**	1
15	1.5	250	28	0.60	**0.71**	0.75	**12**	**8**	0.22	**0.41**	**0.59**	**11**	1
15	1.7	250	24	0.57	0.70	0.75	11	**8**	0.20	0.39	0.49	**11**	0
20	1.5	250	27	0.58	0.68	0.75	11	4	0.24	0.35	0.46	9	0
20	1.7	250	24	**0.61**	0.68	0.74	**12**	4	0.20	0.35	0.44	**11**	0

a For both electrostatic and shape overlap, higher values are better, with 1 indicating perfect overlap. “Min” and “max” columns show the minimum and maximum values, respectively, over the set of ligands. Columns such as “>0.3” indicate the number of ligands that are matched with an overlap greater than the cutoff, with a maximum possible value of 12. In each column, the entry from the best-performing run is shown in bold. The special case of the “repulsive only” FT-FT electrostatic interactions is marked with “R.O” under the *g* column.

### Assessment of Flexible Topology Simulations

To assess the performance of flexible topology pocket exploration (FTPE) sampling conformations, we measure the average similarities between the FTPE frames and conformations of known ligands. Two measures of similarity are used: (1) the spatial overlap and (2) the electrostatic overlap. Both are measured by projecting atomic densities on a common grid after alignment to the 2rh1 template as described in the Alignment and Analysis of Known B2AR Binderssection. The spatial overlap is computed as

6 $$O_{S}\left(x,y\right)=1-\frac{\sum_{i}\left|x_{i}-y_{i}\right|}{\sum_{i}x_{i}+\sum_{i}y_{i}}$$

where *x* and *y* are gridded atomic densities and the sum is over all grid points. This overlap has a maximum of 1 and a minimum of zero. The electrostatic overlap is defined as the root-mean-square difference (RMSD) between two gridded electrostatic potentials. To focus more on qualitative similarities and the general location of positive and negative regions, we use the RMSD between clipped forms of the electrostatic potential (*V*_EP_^c^), which is set to 1 where *V*_EP_ > 0.3, −1 where *V*_EP_ < −0.3, and 0 otherwise. The electrostatic overlap is then computed as

7 $$O_{E}\left(x,y\right)=\frac{2\sum_{i}x_{i}y_{i}}{\sum_{i}\left|x_{i}\right|+\sum_{i}\left|y_{i}\right|}$$

where *x* and *y* are the clipped, gridded electrostatic potentials. Note that *O*_E_ has a theoretical maximum of 1, and *O*_E_ = 0 corresponds to completely uncorrelated maps. *O*_E_ < 0 is possible, and would denote an anticorrelation between the maps.

Table 4 shows the performance of each set of runs in terms of their ability to find frames that match the electrostatic potential and the atomic density as closely as possible for each ligand. Consistent with the earlier analysis, both the simulations with FT-FT electrostatic interaction strengths (*g*) less than 1.2 and the repulsive-only simulations showed poor overlaps, especially in the case of electrostatics. Although many parameter sets showed leading performance in some categories, the set with *N* = 15, *g* = 1.5, and *T*_max_ = 250 stood out, showing leading performance in 6 of the 10 categories examined. Interestingly, the *N* = 20 simulations with *g* = 1.7 and *T*_max_ = 250 were able to demonstrate good spatial overlap (*O*_S_ > 0.6) with all 12 ligands, while the simulations in the *N* = 8 calibration set achieved some of the highest overlap values with the smaller ligands *O*_S_ = 0.79. Performance of each set of runs on each ligand is shown in Tables S1 and S2.

Best electrostatic overlaps are shown in Figure 6, ranging from 0.54 for ALE down to 0.29 for K5Y. Electrostatic overlaps greater than 0.45, as observed for ALE, JTZ, P0G, and CVD, capture the general orientations of the positive and negative isosurfaces. Lower electrostatic overlaps in the range 0.37 to 0.44 showed some similarities, but key components of the ligand isosurfaces were missing. For K5Y (*O*_E_ = 0.29), the closest match did not represent any key aspects of the ligand electrostatics. The best shape overlaps for each ligand are visualized in Figure S3. The spatial envelopes of most ligands are captured well. The ligands with the best agreement are ALE and XQC with shape overlaps of 0.79. Spatial agreement does not generally coincide with electrostatic agreement, as clear differences in charge density can be observed even for ligands with high overlap. There is a weak relationship between the ligand size and shape overlap; the largest ligand (K5Y) was found to have the lowest shape overlap of 0.61.

**Figure 6:**
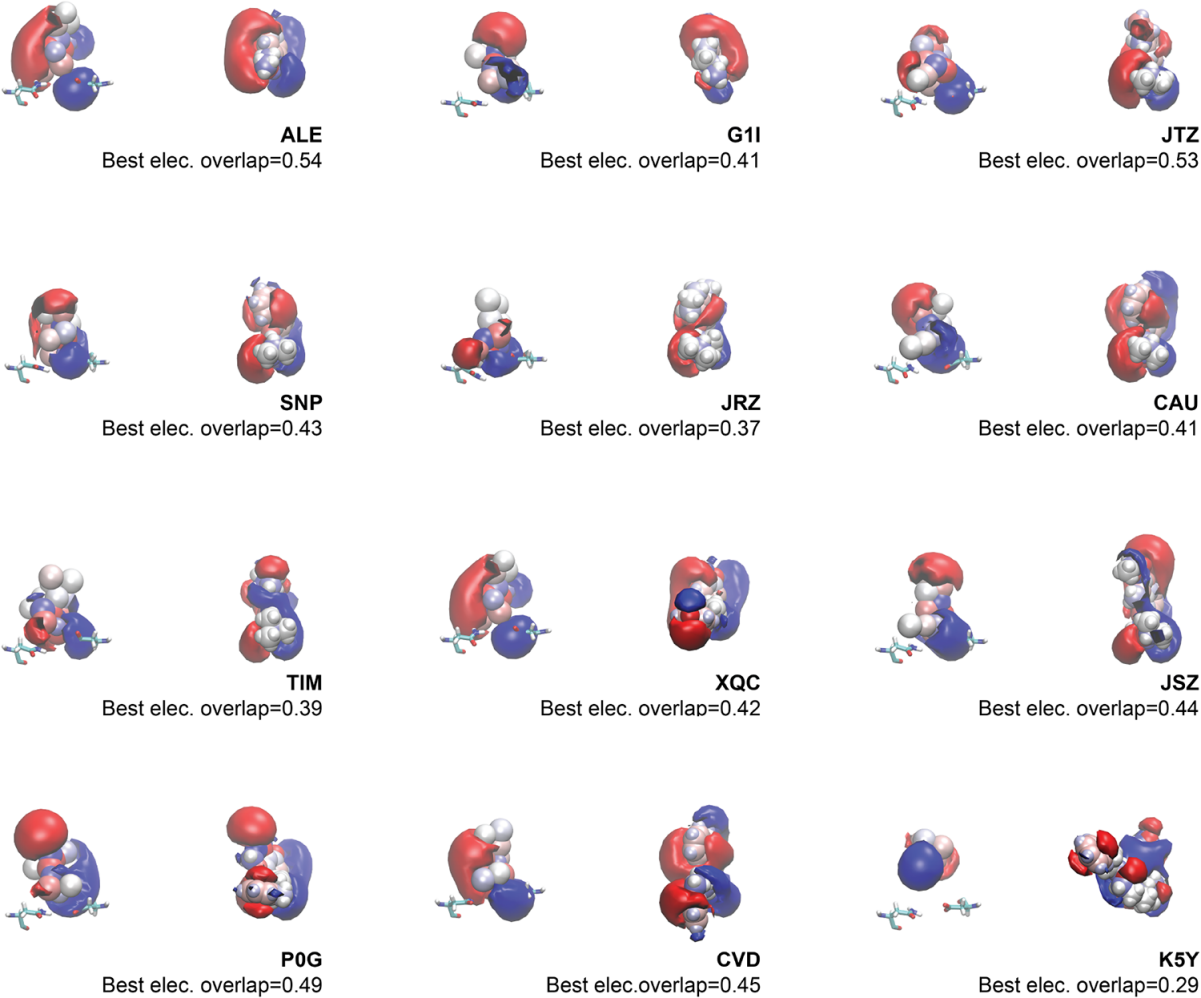
Closest sampled flexible topology conformation to each known ligand in terms of electrostatic overlap. The left half of each panel shows the frame from flexible topology, and the right half shows the ligand. The atoms and electrostatic contours are shown from the exact camera position in each frame, and two residues are shown in the FT frame (Asp113 [right] and Asn312 [left]) for orientation. The electrostatic potential contours are shown using the same cutoff which is 0.3 k for the positive (blue) and negative (red) components. The value of the electrostatic overlap is shown in the bottom right of each panel. The panels are sorted by molecular weight from the lowest to highest.

To analyze the production sets in more detail, we determine the electrostatic potential for each of the 78,500 frames and group them into 500 clusters using the KMeans algorithm. The centroid FT structures corresponding to the 25 most probable clusters are shown in Figure 7, along with the electrostatic potential surfaces. Most of these surfaces (17/25) have a significant positive component (blue) in the vicinity of Asp113. In contrast, other clusters (e.g., 9, 15, 22) appear to have small or negligible volumes of high electrostatic potential. It is likely that these are overemphasized due to the nature of the clustering analysis pipeline, in that regions of low electrostatic density are more similar on average than regions of high electrostatic density. In a comparison of these snapshots with those from known ligands (Figure 3), there are some general similarities in the orientation of the positive and negative components, in the lower region adjacent to Asp113 and Asn312. This demonstrates the ability of FT to extract useful information from the protein binding site. However, the top clusters generally show a weaker negative component (red) and generally exhibit simpler shapes than the electrostatic potential surfaces of the known ligands. The number of frames found in each cluster are shown next to the cluster index. Given that there are 78500 frames in total, there is an average of 157 frames per cluster. All of the top 25 clusters are thus over-represented, ranging from 403 to 297 frames per cluster.

**Figure 7:**
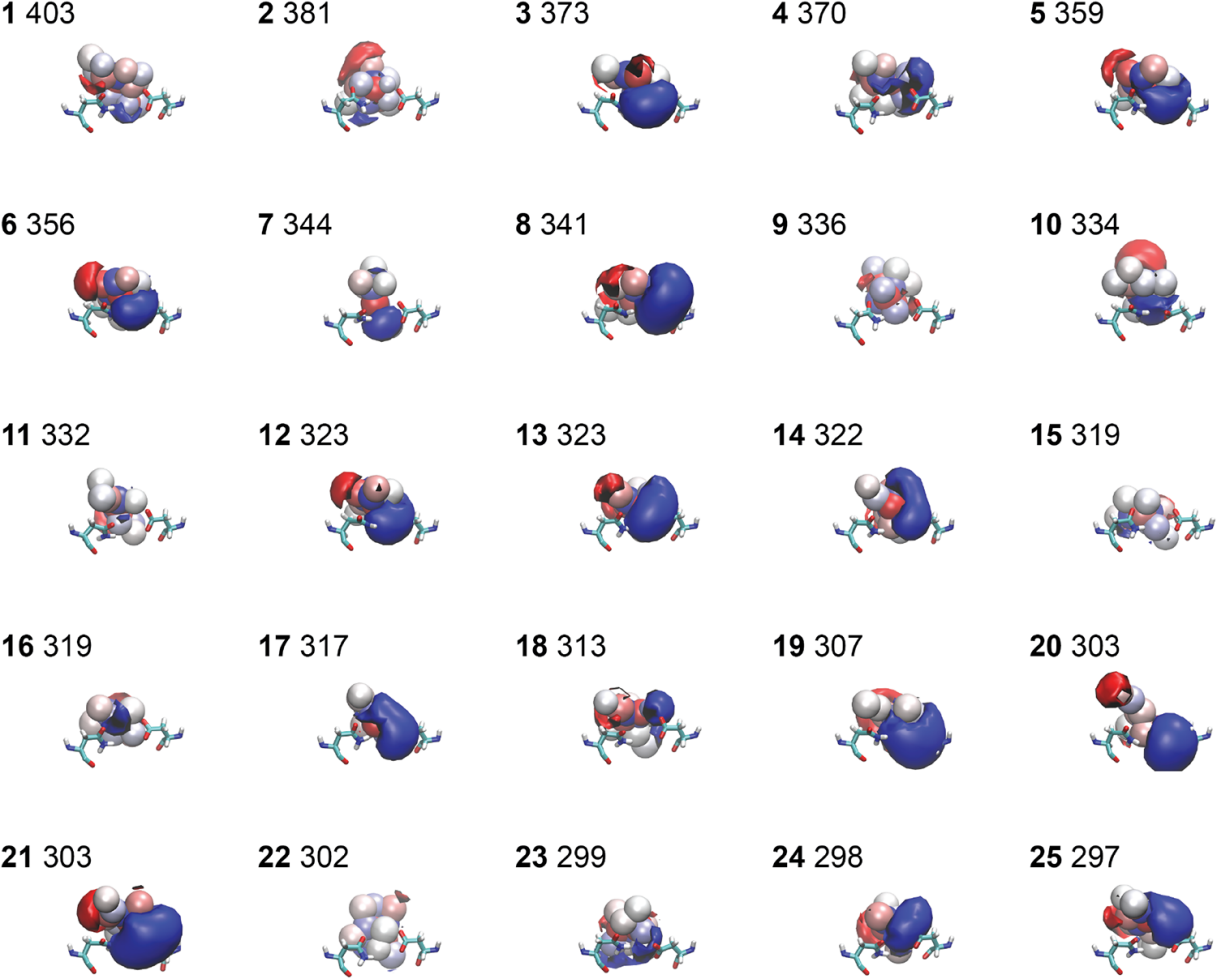
Set of 25 most populated clusters for the production set. Contours are drawn around positive (blue) and negative (red) regions of electrostatic potential that exceed an absolute value of 0.3*k*. All snapshots are taken from the same viewpoint after alignment of the B2AR binding site residues. Asp113 (right) and Asn312 (left) are shown in each frame for the orientation. Cluster labels range from 1 to 25 and are shown in bold. The number of FT conformations in that cluster is shown on the right of the cluster label.

Finally, we examine the prevalence of polar interactions in our data set. Table 5 summarizes the prevalence of polar interactions between the ligands and the binding pocket residues. In the ligand PDB set, the polar interactions were determined by the manual inspection of 28 structures in Table 2. For the FT simulations, every 10th frame was selected for all of the production runs and polar interactions were identified as those in which the electrostatic interaction potential (∑_*i,j*_ *q*_*i*_ *q*_*j*_/*r*_*ij*_) was less than −0.3 e^2^/nm. In the ligand PDB set, polar interactions with Asp113 predominate, being present in almost all structures examined. This is followed by Asn312 (0.387), which acts predominantly as a hydrogen bond donor. Ser203 and Ser207 are present in a smaller fraction of structures and variably act as both acceptors and donors. An Asp192 interaction is present in only PDBID:4ldl. In the set of FT simulations, we again prominently observe Asp113 interactions, although at a slightly lower fraction (0.790). The presence of Asn312 is observed but at much lower likelihood than the PDB set (0.003). We also observe that Lys305 can act as a hydrogen bond donor, which has not been observed in any published structures.

**Table 5:** Comparison of the Prevalence of Polar Interactions for Known Ligands and in Flexible Topology Frames^a^

	residue	role	Frac.
ligand PDBs	Asp113	A	0.968
	Asn312	D/A	0.387
	Ser207	A/D	0.194
	Ser203	A/D	0.161
	Asp192	A	0.032
FT	Asp113	A	0.790
	Lys305	D	0.016
	Asn312	D/A	0.003

a “A” in the Role column denotes hydrogen bond acceptor activity, “D” denotes hydrogen bond donor activity. “D/A” or “A/D” indicates that both donor and acceptor activity are observed, with the predominant role stated first.

### Electrostatics-Based Screening

Given the similarity between electrostatic distributions from Flexible Topology and those of known ligands, a natural question is whether FT data can be used in virtual screening to search for new ligands. We examine two databases in this work: (1) the Human Metabolome Database (HMDB), version 5.0,^34^ containing 220,945 molecules, including those with known activity against B2AR, (2) a subset of the ZINC database, containing approximately 1.2 million drug-like compounds. Each entry in the databases is screened against a query selected from our flexible topology simulations. Specifically, this query was generated using a final temperature of 250 K and a *g* factor of 1.5 and 15 FT particles. The query (Figure 8A) was chosen randomly from a set of frames that showed a polar interaction with Asp113 and had a globularity value of less than 0.2. The shape and electrostatics-based screening tool EON from OpenEye Scientific was used to determine Shape Tanimoto and Charge Tanimoto overlaps between the query and each entry in the database. Each Tanimoto score has a maximum value of 1, resulting in a theoretical maximum combined score of 2.0. The scores are computed irrespective of the number of atoms in each molecule. As shown in Figure 8C, the general shape scores between the ZINC hits and the FT query are reasonably good, corresponding to shape overlap values between 0.65 and 0.70 (Figure 8D). The top hits were also able to capture some electrostatic aspects of the query, including the four strongly charged particles toward the bottom of the molecule. This corresponded to Charge Tanimoto values between 0.42 and 0.47.

**Figure 8:**
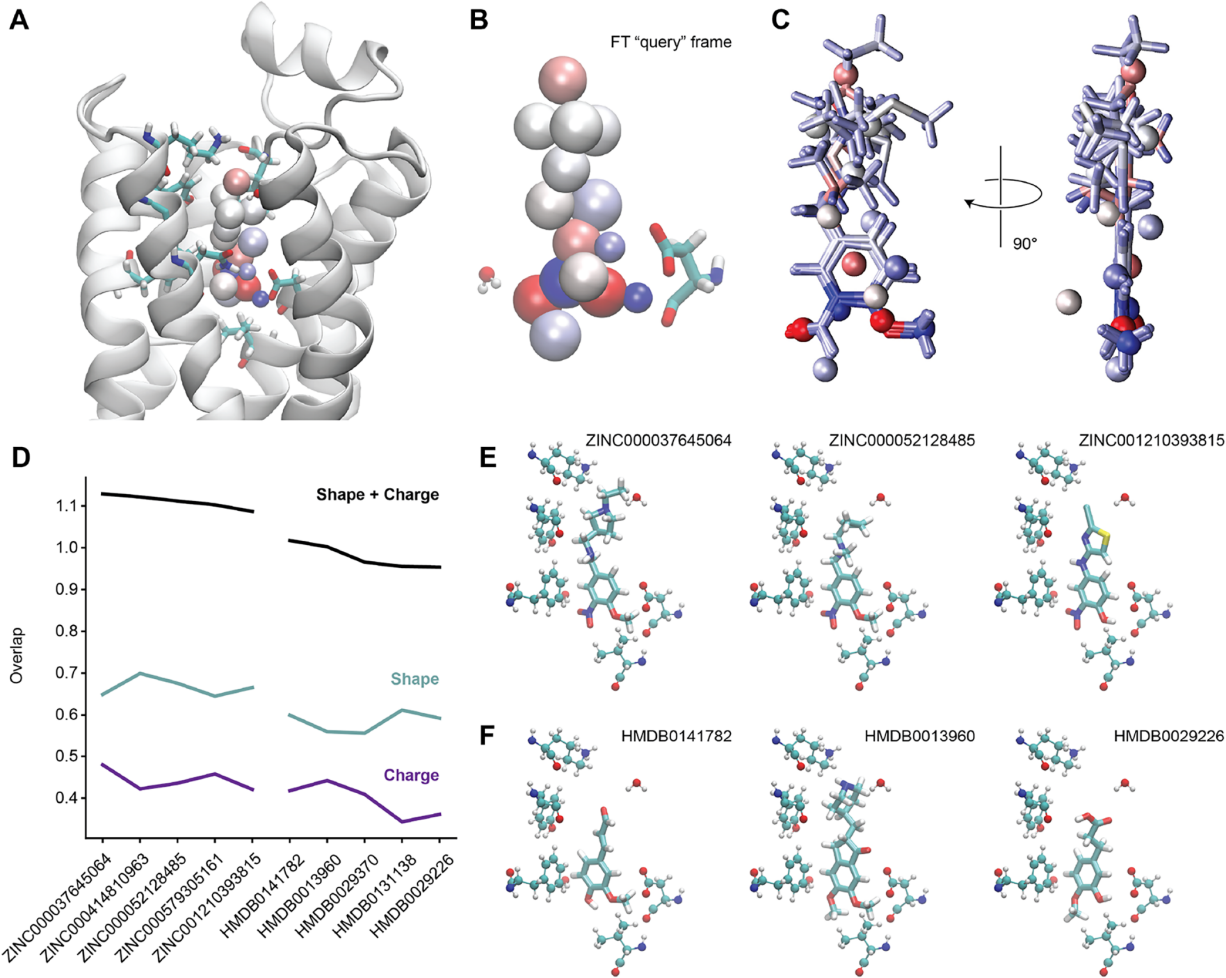
Screening results using a flexible topology frame as a query. (A) The particular FT frame was used as a query as well as surrounding residues. Helix TM1 is omitted for clarity. (B) Details showing interacting residue Asp113 (left) and a binding site water molecule. (C) Hits from the ZINC database overlaid with the FT query. Molecules are shown in licorice and are colored by a partial charge. (D) Overlap values for the top five ZINC hits (left) and top five HMDB hits (right). Hits are ranked by the combined Shape + Charge Tanimoto score (black), which is the sum of the Shape Tanimoto (cyan) and Charge Tanimoto (purple) scores. Individual hits from ZINC (E) and HMDB (F) are shown, along with surrounding B2AR residues and water molecules.

For comparison, we also assessed the overlap of known B2AR ligands with molecules in the ZINC data set (Figure S5). The average best overlap (1.14) was comparable to the best overlap with the FT frame in Figure 8 (1.12). However, the range of best overlaps varied considerably, from 1.29 for JRZ to 0.73 for K5Y. The known ligands mostly showed improvements in both the Shape and Charge Tanimoto scores, with the Shape being >0.70 for 10/13 queries and the Charge being >0.47 for 7/13 queries.

Despite the inclusion of known B2AR actives in the HMDB data set, the top hits showed lower overlaps than observed for ZINC (mean Shape Tanimoto: 0.58, mean Charge Tanimoto: 0.39). This is consistent with the smaller size of the database. The known actives did not appear near the top of this set of hits, and their overlapped poses did not correspond to the crystallographic structure. For example, the highest-scoring known active compound was HMDB0015520 (oxprenolol), with overlaps of 0.60 and 0.20 for Shape and Charge Tanimoto, respectively. While different query frames can result in somewhat higher overlaps with known actives, in general, this approach was not able to pull out known actives from the data set.

Visualization of the top hits in the context of the binding site is shown for ZINC and HMDB in Figure 8E,F. These results are promising in that there is little to no overlap with binding pocket atoms and surrounding waters. In addition, a hydrogen bond acceptor interaction with a binding site water molecule (lower left) was reproduced by all of the top ZINC hits. Hydrogen bond donor activity with Asp113 was observed in some of the hits as well, while in others, the donor hydrogen was replaced by a methyl group.

### Discussion

Here we have used physics-based simulation to build pseudomolecular templates of protein binding ligands for known binding pockets. This is achieved using a set of particles with flexible chemical types with sizes and partial charges that change in response to their surroundings. The dynamical variable formalism used here to model these attributes is similar to that used in the λ-dynamics method,^49^ where the λ parameter typically describes the presence of one or more atoms. As is the case here for σ, ε, and *q*, in λ-dynamics, the equations of motion for λ are determined by partial derivatives of the energy function. By collecting statistics for how often certain λ values are visited, the λ-dynamics method allows for the determination of ligand-binding free energies for sets of prespecified ligands of interest,^50^ while the flexible topology approach is more undirected.

This method also bears similarities to pharmacophore-based methods, in which templates of spatially arranged ligand features (such as “h-bond donor”, “h-bond acceptor”, or “hydrophobic”) are used to rapidly scan large databases for ligands with matching sets of features.^51–53^ Using a charge- and shape-based screening program like EON as a search tool is similar to pharmacophore methods but with a continuous set of ligand features. While a continuous feature search is inherently slower than discrete pharmacophores, it is potentially more expressive as it distinguishes between different strengths of polar hydrogen bond donors and acceptors. That said, it is also possible to directly convert flexible topology frames into discrete pharmacophore models for a more efficient search. Other shape-based tools such as ROCS (OpenEye) can also be applied to FT frames after the discretization of their features.

A variety of restraints and constraints were used to improve the overlap of the FT particles with drug-like ligands. These include constraints on the total charge and the total charge variance, a restraining potential to prevent the particles from breaking into groups (ContForce), and a restraining potential affecting the attributes of each individual particle (AttrForce). A modified “super-charged” Coulomb potential was applied to the interactions between the FT particles to prevent the formation of large dipoles. While the resulting FT particle distributions were found to replicate key interactions with binding site residues of B2AR, overlap with existing and prospective ligands could be further improved. One approach is to replace or augment these ad-hoc restraints with a smarter collective restraining potential using a neural network trained on drug-like ligands. This could collectively bias the particles toward structures that are similar in shape and charge distribution to real compounds, likely improving the overlap with known positives and improving the quality of hits from screens using FT queries.

Although the calibration simulations here provided sets of parameters that showed optimal overlap with the set of known B2AR ligands, this method can also be used on other binding pockets in the absence of any known ligands. A suitable number of particles can be estimated using these results as a guide or determined iteratively by examining unused space or crowding in the binding pocket. It is currently unclear to what extent this knowledge can be transferred to other binding pockets, which will likely have different sizes and tolerances for charge variation. However, a data set of known ligands is by no means required for future applications of this method. In fact, calibrating solely on the set of known ligand structures is likely limiting as this set is not necessarily representative of the true range of ligands that can bind with high affinity. The approach we take here in determining multiple sets of production parameters for analysis is a good way to allow for a broader search of the space of possible ligands.

The most unique aspect of this approach is its ability to simultaneously sample different ligand morphologies and pocket shapes. A key element of the FT results, aside from the FT particle distributions themselves, is the coordinates of the surrounding protein and waters. Figure 8 shows how these coordinates can be used to model hit molecules in the binding site. These can also inform subsequent docking calculations or act as starting points for free energy calculations. The ability to sample pocket shapes that are consistent with the presence of a ligand will likely be most useful in scenarios where the binding pocket is flexible and/or the conformation of the binding pocket residues is unknown. Nuclear receptors such as RXR are examples of highly flexible receptors, where ligand binding can initiate different oligomeric states and functions. Novel target classes such as protein–protein interaction inhibitors and molecular glues could benefit from this approach, as the binding pockets are often nonphysiological, and the orientation of the binding pocket residues is unknown.

The flexible topology pocket explorer (FTPE) method could also be powerful when used comparatively. Sets of FTPE simulations could be run in multiple environments, corresponding to either different receptor subtypes or different conformations. Using a method like linear discriminant analysis to focus on the differences in FT distributions could help identify particular queries that show maximum selectivity between the environments.

## Supplementary Material



## Notes

Scatter plots showing the relationship between nonbonded parameters in a large chemical data set (Figure S1); electrostatic potential surfaces for each individual ligand (top view) (Figure S2); closest sampled flexible topology conformation to each known ligand in terms of shape overlap (Figure S3); backbone root-mean-squared deviation of the protein for 18 ns FT simulations (Figure S4); overlaps for the best matching molecules in our ZINC data set with known ligand queries (Figure S5); performance of each run in finding the matching the shape for each known ligand (Table S1); and performance of each run in finding the matching the electrostatics for each known ligand (Table S2) (PDF)

## References

[1] Ruzmetov, T. ; Hung, T. I. ; Jonnalagedda, S. P. ; Chen, S.-h. ; Guo, Z. ; Bhanu, B. ; Chang, C.-e. A. Sampling Conformational Ensembles of Highly Dynamic Proteins via Generative Deep Learning Comput. Chem., 2487 2502 10.1021/acs.jcim.4c01838. PMC13401506 39984300

[2] Lane T. J. (2023). Protein
structure prediction has reached the single-structure frontier. Nat. Methods.

[3] Lu W., Zhang J., Huang W., Zhang Z., Jia X., Wang Z., Shi L., Li C., Wolynes P. G., Zheng S. (2024). DynamicBind: predicting ligand-specific protein-ligand complex structure
with a deep equivariant generative model. Nat.
Commun..

[4] Nussinov R., Zhang M., Liu Y., Jang H. (2023). AlphaFold, allosteric,
and orthosteric drug discovery: Ways forward. Drug Discovery Today.

[5] Hekkelman M. L., de Vries I., Joosten R. P., Perrakis A. (2023). AlphaFill:
enriching
AlphaFold models with ligands and cofactors. Nat. Methods.

[6] Zhou G., Rusnac D., Park H., Canzani D., Nguyen H. M., Stewart L., Bush M. F., Nguyen P. T., Wulff H., Yarov-Yarovoy V., Zheng N., Dimaio F. (2024). An artificial intelligence
accelerated virtual screening platform for drug discovery. Nat. Commun..

[7] Rational Drug Design. In Methods and Protocols; Mavromoustakos, T. ; Kellici, T. F. , Eds.; Methods in Molecular Biology; Springer New York: New York, NY, 2018; Vol. 1824.

[8] Teague S. J. (2003). Implications
of protein flexibility for drug discovery. Nat.
Rev. Drug Discovery.

[9] Han, Z. ; Panhans, S. ; Bilgen, E. Dissecting Mechanisms of Ligand Binding and Conformational Changes in the Glutamine-Binding Protein eLife 2024; Vol. 13. 10.7554/eLife.95304 PMC13229503 42227972

[10] Toporowska J., Kapoor P., Musgaard M., Gherbi K., Sengmany K., Qu F., Soave M., Yen H.-Y., Hansen K., Jazayeri A., Hopper J. T. S., Politis A. (2024). Ligand-induced conformational changes
in the Beta1-adrenergic receptor revealed by hydrogen-deuterium exchange
mass spectrometry. Nat. Commun..

[11] Khan S. H., Braet S. M., Koehler S. J., Elacqua E., Anand G. S., Okafor C. D. (2022). Ligand-induced shifts in conformational
ensembles that
describe transcriptional activation. eLife.

[12] Cimermancic P., Weinkam P., Rettenmaier T. J., Bichmann L., Keedy D. A., Woldeyes R. A., Schneidman-Duhovny D., Demerdash O. N., Mitchell J. C., Wells J. A., Fraser J. S., Sali A. (2016). CryptoSite:
Expanding the Druggable Proteome by Characterization and Prediction
of Cryptic Binding Sites. J. Mol. Biol..

[13] Baron R., Setny P., McCammon J. A. (2010). Water in
Cavity-Ligand Recognition. J. Am. Chem. Soc..

[14] Whitesides G. M., Krishnamurthy V. M. (2005). Designing
ligands to bind proteins. Q. Rev. Biophys..

[15] Hu B., Lill M. A. (2012). Protein Pharmacophore
Selection Using Hydration-Site
Analysis. J. Chem. Inf. Model..

[16] Kumar P., Mohanty D. (2022). Development of a Novel
Pharmacophore Model Guided by
the Ensemble of Waters and Small Molecule Fragments Bound to SARS-CoV-2
Main Protease. Mol. Inf..

[17] Raymer M. L., Sanschagrin P. C., Punch W. F., Venkataraman S., Goodman E. D., a Kuhn L. (1997). Predicting conserved water-mediated
and polar ligand interactions in proteins using a K-nearest-neighbors
genetic algorithm. J. Mol. Biol..

[18] Schiebel J., Gaspari R., Wulsdorf T., Ngo K., Sohn C., Schrader T. E., Cavalli A., Ostermann A., Heine A., Klebe G. (2018). Intriguing role of water in protein-ligand
binding studied by neutron crystallography on trypsin complexes. Nat. Commun..

[19] Giordano D., Biancaniello C., Argenio M. A., Facchiano A. (2022). Drug Design
by Pharmacophore and Virtual Screening Approach. Pharmaceuticals.

[20] Tang Y., Moretti R., Meiler J. (2024). Recent Advances in Automated Structure-Based
De Novo Drug Design. J. Chem. Inf. Model..

[21] Therrien E., Weill N., Tomberg A., Corbeil C. R., Lee D., Moitessier N. (2014). Docking Ligands
into Flexible and Solvated Macromolecules.
7. Impact of Protein Flexibility and Water Molecules on Docking-Based
Virtual Screening Accuracy. J. Chem. Inf. Model..

[22] Park H., Zhou G., Baek M., Baker D., DiMaio F. (2021). Force Field
Optimization Guided by Small Molecule Crystal Lattice Data Enables
Consistent Sub-Angstrom Protein-Ligand Docking. J. Chem. Theory Comput..

[23] Paggi J. M., Pandit A., Dror R. O. (2024). The Art and Science
of Molecular
Docking. Annu. Rev. Biochem..

[24] Nada H., Meanwell N. A., Gabr M. T. (2025). Virtual
screening: hope, hype, and
the fine line in between. Expert Opin. Drug
Discovery.

[25] Basciu, A. ; Athar, M. ; Kurt, H. ; Neville, C. ; Malloci, G. ; Muredda, F. C. ; Bosin, A. ; Ruggerone, P. ; Bonvin, A. M. J. J. ; Vargiu, A. V. Predicting binding events in very flexible, allosteric, multi-domain proteins bioRxiv 2024 https://www.biorxiv.org/content 10.1101/2024.06.02.597018v1. PMC11863385 39907634

[26] Donyapour N., Niazi F. F., Roussey N. M., Bose S., Dickson A. (2023). Flexible Topology:
A Dynamic Model of a Continuous Chemical Space. J. Chem. Theory Comput..

[27] Behler J., Parrinello M. (2007). Generalized neural-network representation
of high-dimensional
potential-energy surfaces. Phys. Rev. Lett..

[28] Xing R.-J., Wang J., Pan L., Cheng M.-S. (2009). A Selective Pharmacophore
Model for Beta2-Adrenoceptor Agonists. Molecules.

[29] Cherezov V., Rosenbaum D. M., Hanson M. A., Rasmussen S. G. F., Thian F. S., Kobilka T. S., Choi H.-J., Kuhn P., Weis W. I., Kobilka B. K., Stevens R. C. (2007). High Resolution
Crystal Structure of an Engineered Human Beta2-Adrenergic G protein-Coupled
Receptor. Science.

[30] Parichatikanond W., Duangrat R., Kurose H., Mangmool S. (2024). Regulation of Beta-Adrenergic
Receptors in the Heart: A Review on Emerging Therapeutic Strategies
for Heart Failure. Cells.

[31] McGraw D. W., Liggett S. B. (2005). Molecular Mechanisms
of Beta2-Adrenergic Receptor Function
and Regulation. Proc. Am. Thorac. Soc..

[32] Tashkin D. P., Fabbri L. M. (2010). Long-acting beta-agonists
in the management of chronic
obstructive pulmonary disease: current and future agents. Respir. Res..

[33] Irwin J. J., Tang K. G., Young J., Dandarchuluun C., Wong B. R., Khurelbaatar M., Moroz Y. S., Mayfield J., Sayle R. A. (2020). ZINC20 - A Free
Ultralarge-Scale Chemical Database
for Ligand Discovery. J. Chem. Inf. Model..

[34] Wishart D. S., Guo A., Oler E. (2022). HMDB 5.0: the Human Metabolome Database for
2022. Nucleic Acids Res..

[35] Jo S., Kim T., Iyer V. G., Im W. (2008). CHARMM-GUI: A web-based graphical
user interface for CHARMM. J. Comput. Chem..

[36] Ring A. M., Manglik A., Kruse A. C., Enos M. D., Weis W. I., Garcia K. C., Kobilka B. K. (2013). Adrenaline-activated
structure of
Beta2-adrenoceptor stabilized by an engineered nanobody. Nature.

[37] Lomize M. A., Pogozheva I. D., Joo H., Mosberg H. I., Lomize A. L. (2012). OPM database
and PPM web server: resources for positioning of proteins in membranes. Nucleic Acids Res..

[38] Ingólfsson H. I., Melo M. N., Eerden F. J. V., Arnarez C., Lopez C. A., Wassenaar T. A., Periole X., Vries A. H. D., Tieleman D. P., Marrink S. J. (2014). Lipid organization of the plasma membrane. J. Am. Chem. Soc..

[39] Eastman P., Galvelis R., Peláez R. P. (2024). OpenMM 8: Molecular
Dynamics Simulation with Machine Learning Potentials. J. Phys. Chem. B.

[40] Donyapour, N. ; Roussey, N. M. ; Dickson, A. ; Niazi, F. F. FlexibleTopology. https://github.com/ADicksonLab/flexibletopology.

[41] Openmmcontinuityforce: An OpenMM force that ensures a set of atoms stay continuously connected in space. https://github.com/alexrd/openmmcontinuityforce.

[42] Donyapour N., Hirn M., Dickson A. (2021). ClassicalGSG:
Prediction of log P
using classical molecular force fields and geometric scattering for
graphs. J. Comput. Chem..

[43] Korshunova M., Ginsburg B., Tropsha A., Isayev O. (2021). OpenChem: ADeep Learning
Toolkit for Computational Chemistry and Drug Design. J. Chem. Inf. Model..

[44] Vanommeslaeghe K., Hatcher E., Acharya C., Kundu S., Zhong S., Shim J., Darian E., Guvench O., Lopes P., Vorobyov I., Mackerell A. D. (2010). CHARMM General
Force Field (CGenFF): A force field for drug-like molecules compatible
with the CHARMM all-atom additive biological force fields. J. Comput. Chem..

[45] Pdbfixer: PDBFixer fixes problems in PDB files. https://github.com/openmm/pdbfixer.

[46] Boothroyd S., Behara P. K., Madin O. C. (2023). Development and Benchmarking
of Open Force Field 2.0.0: The Sage Small Molecule Force Field. J. Chem. Theory Comput..

[47] O’Boyle N. M., Banck M., James C. A., Morley C., Vandermeersch T., Hutchison G. R. (2011). Open Babel: An Open chemical toolbox. J. Cheminf..

[48] Halgren T. A. (1996). Merck molecular
force field. I. Basis, form, scope, parameterization, and performance
of MMFF94. J. Comput. Chem..

[49] Guo Z., Brooks C. L. (1998). Rapid screening
of binding affinities: Application
of the lambda-dynamics method to a trypsin-inhibitor system. J. Am. Chem. Soc..

[50] Raman E. P., Paul T. J., Hayes R. L., B C.
L. (2020). Automated, Accurate, and Scalable Relative Protein-Ligand Binding
Free-Energy Calculations Using Lambda Dynamics. J. Chem. Theory Comput..

[51] Meagher K. L., Carlson H. A. (2004). Incorporating protein flexibility in structure-based
drug discovery: using HIV-1 protease as a test case. J. Am. Chem. Soc..

[52] Hu B., Lill M. A. (2012). Protein pharmacophore selection using hydration-site
analysis. J. Chem. Inf. Model..

[53] Sunseri J., Koes D. R. (2016). Pharmit: interactive
exploration of chemical space. Nucleic Acids
Res..

