# Supplementary material for: Undirected Exploration of Binding Pockets with Flexible Topology
Source: J Chem Theory Comput. 2025 Oct 9;21(20):10398–412. doi: 10.1021/acs.jctc.5c00825 (PMC12573749; doi:10.1021/acs.jctc.5c00825)
Supplement: Supplementary file 1 [file ct5c00825_si_001.pdf]

Supplementary Information: Undirected exploration of binding pockets with Flexible Topology

Fatemeh Fathi Niazi,† Seungmin Yoon,‡ Khadim Mbacke,¶ and Alex Dickson*,†,§

†*Department of Computational Mathematics, Science and Engineering, Michigan State University, East Lansing, Michigan 48824, USA*

‡*Department of Pharmacology and Toxicology, Michigan State University, East Lansing, Michigan 48824, USA*

¶*Department of Physics and Astronomy, Michigan State University, East Lansing, Michigan 48824, USA*

§*Department of Biochemistry and Molecular Biology, Michigan State University, East Lansing, Michigan 48824, USA*

E-mail: alexrd@msu.edu

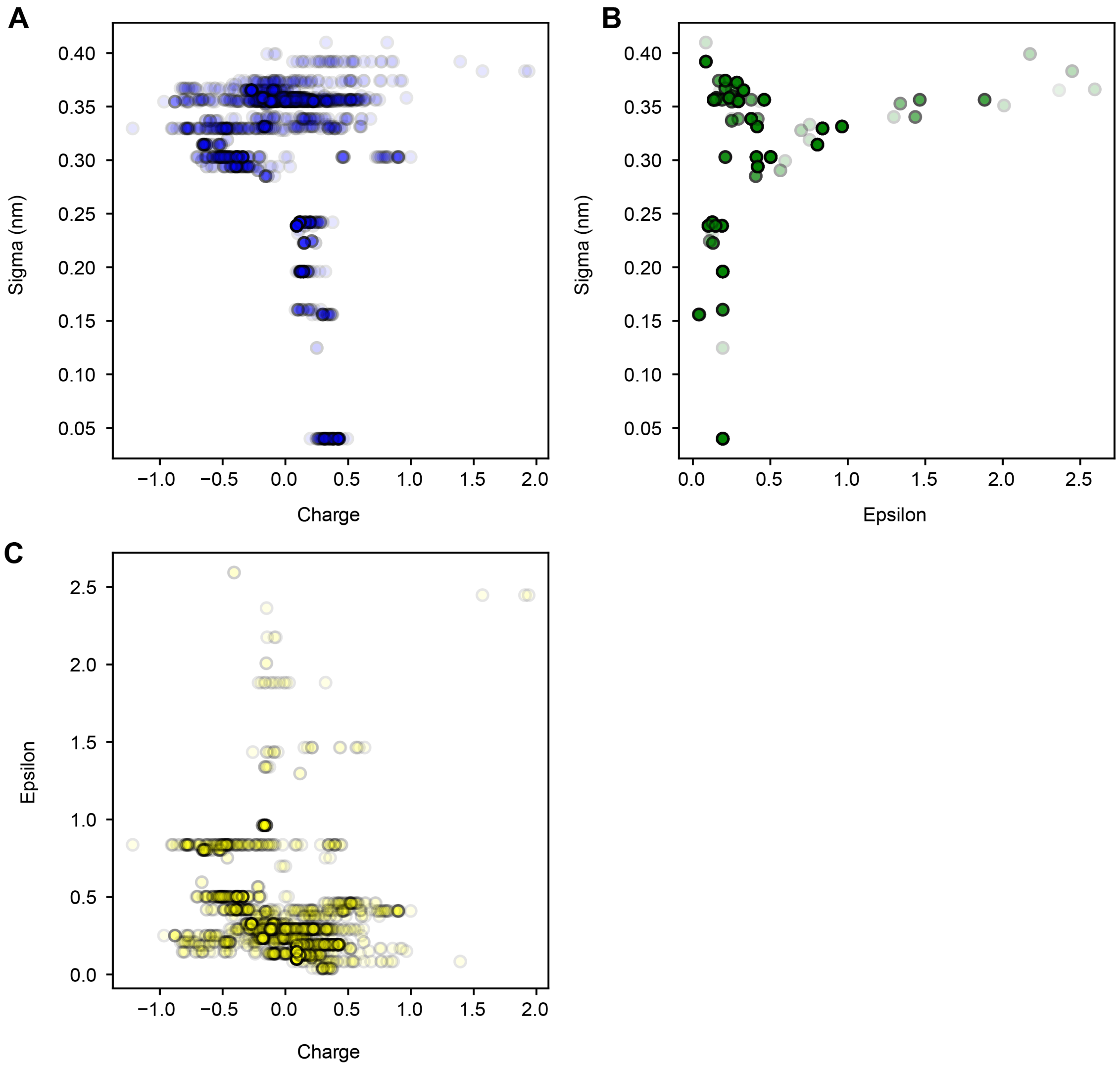

Figure S1: Relationships between atomic attributes as examined on a large chemical dataset. Individual points are shown as transparent symbols, although most parameter combinations are observed many times in the dataset.

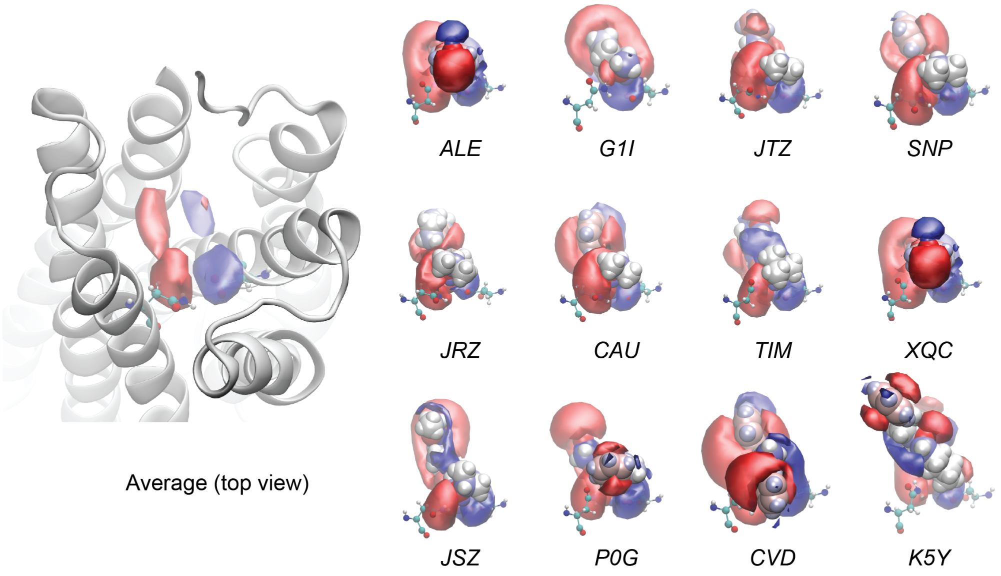

Figure S2: Electrostatic potential surfaces for each individual ligand (top view). Positive (blue) and negative (red) regions are shown using isovalues of $0.3k$ and $-0.3k$ respectively, where k is the Coulomb constant. Ligands are shown in size-charge representation, along with two B2AR binding pocket residues in CPK representation (Asp113 [right] and Asn312 [left]). The snapshots of the average and the individual ligands were taken using the same camera orientation after alignment of binding pocket residues.

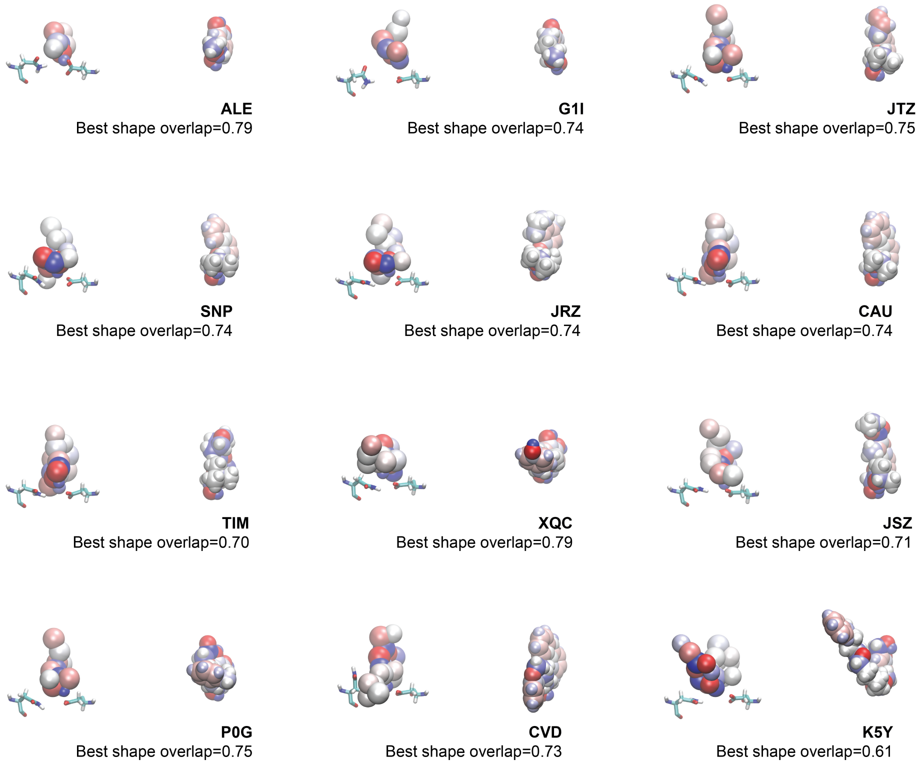

Figure S3: Closest sampled flexible topology conformation to each known ligand in terms of shape overlap. The left half of each panel shows the frame from flexible topology, the right half shows the ligand. The atoms are all shown from the same camera position in each frame and two residues are shown in the FT frame (Asp113 [right] and Asn312 [left]) for orientation. The value of the shape overlap is shown in the bottom right of each panel. The panels are sorted by molecular weight from lowest to highest.

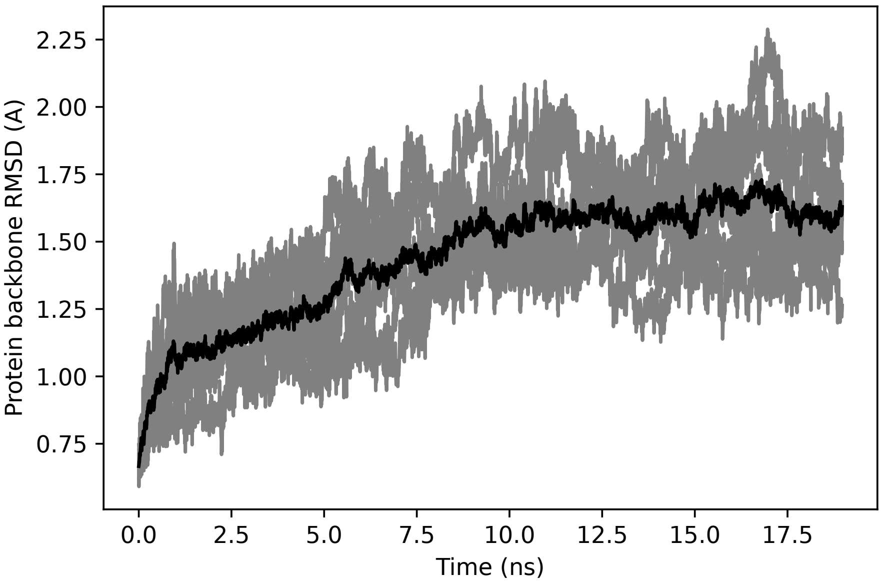

Figure S4: The protein stability is examined for a set of eight longer FT simulations (18 ns), using the backbone root mean squared deviation of the protein. Individual curves are shown in grey, with the average over the set shown in black. RMSD is computed with respect to the initial structure and $t = 0$ corresponds to the end of the heating phase. All simulations were conducted with $N = 15$ particles, $g = 1.7$ and $T = 300$. The backbone RMSD was roughly constant during the last half of the simulations, plateauing at a value of 1.60 Å.

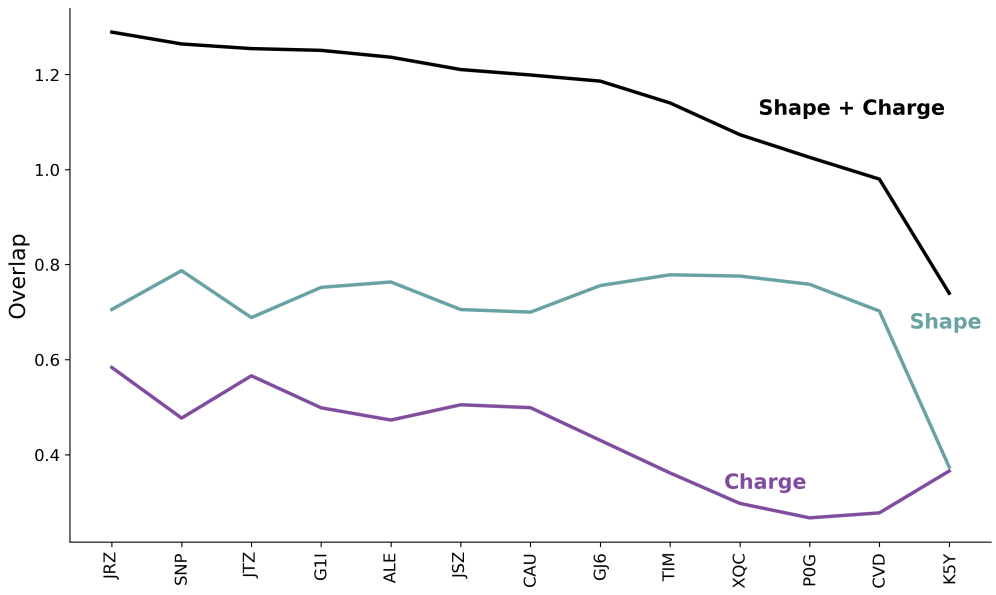

Figure S5: Overlaps for the best matching molecules in our ZINC dataset with known ligand queries.

Set details				ALE		G1I		JTZ		SNP	
N	g	max T	Nruns	mean	max	mean	max	mean	max	mean	max
8	1.7	300	5	0.47	**0.79**	0.49	0.73	0.46	0.67	0.45	0.62
10	1.7	300	5	0.41	0.73	0.44	**0.74**	0.43	0.68	0.42	0.68
12	1.7	300	5	0.52	0.75	**0.52**	0.72	0.50	0.73	0.48	0.68
15	1.7	300	5	0.41	0.65	0.41	0.66	0.42	0.67	0.43	0.70
20	1.7	300	3	0.37	0.56	0.39	0.59	0.39	0.61	0.40	0.62
15	0.0	300	5	0.17	0.39	0.15	0.36	0.18	0.35	0.19	0.35
15	R.O.	300	5	0.34	0.57	0.32	0.56	0.37	0.62	0.37	0.62
15	0.8	300	5	0.28	0.63	0.26	0.60	0.32	0.67	0.32	0.67
15	1.0	300	5	0.42	0.63	0.38	0.65	0.40	0.66	0.43	0.71
15	1.2	300	5	0.46	0.65	0.46	0.67	0.45	0.70	0.46	0.69
15	1.5	300	5	0.50	0.68	0.50	0.69	0.49	0.71	0.49	0.67
15	2.0	300	5	0.45	0.69	0.45	0.70	0.45	0.62	0.45	0.67
15	2.5	300	5	0.44	0.68	0.47	0.68	0.47	0.69	0.46	0.68
15	3.0	300	5	0.42	0.63	0.41	0.68	0.41	0.61	0.43	0.64
15	1.5	200	7	0.51	0.68	0.50	0.69	**0.54**	0.74	**0.53**	0.70
15	1.5	250	7	0.51	0.69	0.47	0.67	0.52	0.70	**0.53**	0.72
12	1.5	250	28	0.50	0.74	0.48	0.72	0.48	0.74	0.49	0.73
12	1.7	250	26	**0.53**	0.76	0.50	0.73	0.50	0.73	0.49	0.72
15	1.5	250	28	0.50	0.69	0.50	0.71	0.53	**0.75**	0.52	**0.74**
15	1.7	250	24	0.51	0.72	0.49	0.70	0.53	0.74	**0.53**	**0.74**
20	1.5	250	27	0.45	0.62	0.44	0.63	0.50	0.70	0.51	0.70
20	1.7	250	24	0.46	0.62	0.45	0.61	0.51	0.70	0.51	0.72

Set details				JRZ		CAU		TIM		XQC	
N	g	max T	Nruns	mean	max	mean	max	mean	max	mean	max
8	1.7	300	5	0.40	0.58	0.45	0.63	0.42	0.57	0.37	0.56
10	1.7	300	5	0.41	0.61	0.42	0.64	0.39	0.60	0.37	0.67
12	1.7	300	5	0.47	0.67	0.49	0.69	0.45	0.64	0.46	0.71
15	1.7	300	5	0.45	0.70	0.41	0.67	0.38	0.62	0.46	0.70
20	1.7	300	3	0.46	0.64	0.40	0.61	0.35	0.55	0.49	0.70
15	0.0	300	5	0.18	0.34	0.17	0.32	0.18	0.28	0.22	0.47
15	R.O.	300	5	0.38	0.59	0.38	0.62	0.34	0.55	0.38	0.65
15	0.8	300	5	0.32	0.64	0.31	0.68	0.29	0.60	0.38	0.70
15	1.0	300	5	0.42	0.62	0.38	0.63	0.37	0.61	0.54	0.72
15	1.2	300	5	0.46	0.67	0.43	0.68	0.41	0.62	0.55	**0.79**
15	1.5	300	5	0.50	0.68	0.47	0.67	0.43	0.64	0.55	0.75
15	2.0	300	5	0.47	0.69	0.44	0.62	0.42	0.59	0.49	0.70
15	2.5	300	5	0.49	0.66	0.48	0.69	0.43	0.63	0.43	0.76
15	3.0	300	5	0.46	0.62	0.41	0.60	0.38	0.56	0.46	0.68
15	1.5	200	7	0.52	0.68	0.50	0.70	0.46	0.64	0.56	0.74
15	1.5	250	7	0.53	0.73	0.49	0.70	0.46	0.63	**0.57**	0.76
12	1.5	250	28	0.49	0.71	0.45	0.68	0.42	0.67	0.51	0.73
12	1.7	250	26	0.50	0.71	0.47	0.70	0.45	**0.70**	0.51	0.72
15	1.5	250	28	0.52	**0.74**	0.49	**0.74**	0.47	**0.70**	0.56	0.75
15	1.7	250	24	**0.54**	0.72	**0.51**	0.72	**0.48**	0.66	0.56	0.75
20	1.5	250	27	0.50	0.69	0.47	0.69	0.45	0.67	0.56	0.74
20	1.7	250	24	0.51	0.69	0.48	0.69	0.46	0.68	**0.57**	0.74

Set details				JSZ		P0G		CVD		K5Y	
N	g	max T	Nruns	mean	max	mean	max	mean	max	mean	max
8	1.7	300	5	0.42	0.59	0.40	0.52	0.37	0.50	0.29	0.45
10	1.7	300	5	0.41	0.61	0.40	0.60	0.40	0.62	0.31	0.49
12	1.7	300	5	0.47	0.65	0.47	0.63	0.43	0.59	0.35	0.50
15	1.7	300	5	0.39	0.62	0.46	0.71	0.46	0.65	0.37	0.58
20	1.7	300	3	0.40	0.58	0.49	0.72	0.46	0.63	0.43	**0.61**
15	0.0	300	5	0.17	0.41	0.20	0.39	0.17	0.35	0.18	0.42
15	R.O.	300	5	0.41	0.66	0.37	0.59	0.37	0.62	0.36	0.55
15	0.8	300	5	0.30	0.69	0.35	0.64	0.33	0.65	0.33	0.56
15	1.0	300	5	0.37	0.60	0.49	0.72	0.49	0.66	0.39	0.55
15	1.2	300	5	0.42	0.64	0.53	0.74	0.49	0.64	0.42	0.58
15	1.5	300	5	0.46	0.68	0.53	**0.75**	0.47	0.63	0.40	0.55
15	2.0	300	5	0.43	0.59	0.49	0.69	0.47	0.65	0.37	0.53
15	2.5	300	5	0.49	0.67	0.47	0.72	0.46	0.61	0.36	0.54
15	3.0	300	5	0.41	0.60	0.47	0.69	0.47	0.66	0.34	0.54
15	1.5	200	7	**0.50**	**0.71**	0.56	0.74	0.50	0.65	0.43	0.56
15	1.5	250	7	0.48	0.65	0.56	0.73	0.51	0.65	0.42	0.58
12	1.5	250	28	0.44	0.68	0.50	0.71	0.45	0.68	0.39	0.57
12	1.7	250	26	0.45	0.68	0.50	0.68	0.45	0.61	0.38	0.55
15	1.5	250	28	0.49	0.70	0.55	0.74	0.50	0.68	0.41	0.60
15	1.7	250	24	0.49	0.68	0.56	0.73	0.49	0.65	0.42	0.57
20	1.5	250	27	0.47	0.69	**0.57**	**0.75**	**0.53**	**0.73**	0.44	0.58
20	1.7	250	24	0.48	0.66	**0.57**	0.74	**0.53**	0.68	**0.45**	**0.61**

Table S1: Performance of each run in finding the matching the shape for each known ligand. All values shown are shape overlaps (O_S); higher values are better, with 1 indicating perfect overlap. "Mean" and "max" columns show the mean and maximum values, respectively, over the set of FT frames. In each column, the entry from the best performing set is shown in bold. The special case of the "repulsive only" FT-FT electrostatic interactions is marked with "R.O" under the g column.

Set details				ALE		G1I		JTZ		SNP	
N	g	max T	Nruns	mean	max	mean	max	mean	max	mean	max
8	1.7	300	5	0.09	0.52	0.07	0.36	0.07	0.47	0.07	0.38
10	1.7	300	5	0.06	0.42	0.05	0.37	0.04	0.44	0.05	0.37
12	1.7	300	5	0.16	0.53	0.10	0.35	0.17	**0.53**	0.14	0.39
15	1.7	300	5	0.06	0.46	0.04	0.25	0.05	0.46	0.05	0.38
20	1.7	300	3	0.04	0.30	0.02	0.23	0.04	0.34	0.04	0.32
15	0.0	300	5	-0.01	0.02	-0.00	0.01	-0.01	0.00	-0.01	0.00
15	R.O.	300	5	-0.01	0.03	-0.00	0.01	0.00	0.03	-0.00	0.02
15	0.8	300	5	-0.00	0.12	-0.01	0.03	-0.00	0.07	-0.00	0.06
15	1.0	300	5	0.03	0.33	0.00	0.17	0.03	0.25	0.02	0.19
15	1.2	300	5	0.11	0.41	0.05	0.33	0.09	0.33	0.07	0.32
15	1.5	300	5	**0.17**	**0.54**	0.08	0.40	**0.18**	0.52	**0.15**	0.40
15	2.0	300	5	0.09	0.40	0.05	0.33	0.08	0.42	0.07	0.38
15	2.5	300	5	0.04	0.35	0.03	0.29	0.04	0.34	0.04	0.33
15	3.0	300	5	0.05	0.37	0.03	0.26	0.06	0.44	0.05	0.40
15	1.5	200	7	0.16	0.41	0.07	0.29	0.16	0.40	0.12	0.31
15	1.5	250	7	0.16	0.43	0.08	0.35	**0.18**	0.49	0.13	0.40
12	1.5	250	28	0.13	0.49	0.08	0.32	0.13	0.47	0.10	0.41
12	1.7	250	26	0.15	0.51	**0.11**	**0.41**	0.15	0.50	0.14	**0.43**
20	1.5	250	28	0.14	0.59	0.09	0.38	0.15	0.49	0.12	0.40
20	1.7	250	24	0.13	0.49	0.07	0.38	0.14	0.48	0.12	0.42
20	1.5	250	27	0.11	0.46	0.04	0.28	0.12	0.39	0.08	0.36
20	1.7	250	24	0.12	0.42	0.06	0.32	0.14	0.44	0.10	0.34

Set details				JRZ		CAU		TIM		XQC	
N	g	max T	Nruns	mean	max	mean	max	mean	max	mean	max
8	1.7	300	5	0.07	0.35	0.07	0.36	0.05	0.38	0.07	0.40
10	1.7	300	5	0.05	0.29	0.05	0.35	0.05	0.33	0.07	0.35
12	1.7	300	5	0.11	0.32	0.11	0.35	0.12	0.35	0.13	0.38
15	1.7	300	5	0.03	0.26	0.02	0.30	0.05	0.34	0.08	0.34
20	1.7	300	3	0.03	0.23	0.01	0.24	0.04	0.27	0.05	0.25
15	0.0	300	5	-0.01	0.00	-0.01	0.01	0.01	0.01	-0.00	0.01
15	R.O.	300	5	-0.01	0.01	-0.00	0.03	0.00	0.01	0.00	0.03
15	0.8	300	5	-0.01	0.04	-0.00	0.09	0.00	0.04	0.01	0.12
15	1.0	300	5	-0.00	0.16	0.02	0.22	0.02	0.16	0.05	0.31
15	1.2	300	5	0.04	0.25	0.06	0.27	0.06	0.27	0.11	0.34
15	1.5	300	5	0.10	0.29	**0.12**	0.39	**0.13**	0.35	**0.15**	0.39
15	2.0	300	5	0.07	0.27	0.05	0.31	0.07	0.32	0.08	0.30
15	2.5	300	5	0.05	0.30	0.03	0.26	0.03	0.29	0.04	0.31
15	3.0	300	5	0.05	0.28	0.03	0.35	0.03	0.33	0.04	0.30
15	1.5	200	7	0.09	0.27	0.11	0.30	0.11	0.27	0.13	0.31
15	1.5	250	7	0.08	0.29	0.10	0.33	**0.13**	0.36	0.14	0.31
12	1.5	250	28	0.09	0.33	0.07	0.35	0.08	0.32	0.12	0.36
12	1.7	250	26	**0.12**	**0.37**	0.10	**0.41**	0.11	0.37	0.13	0.39
15	1.5	250	28	0.09	0.34	0.10	0.35	0.12	**0.39**	0.13	**0.42**
15	1.7	250	24	0.09	0.33	0.09	0.37	0.11	0.36	0.11	0.37
20	1.5	250	27	0.05	0.26	0.07	0.32	0.09	0.31	0.12	0.35
20	1.7	250	24	0.08	0.32	0.07	0.32	0.11	0.36	0.11	0.35

Set details				JSZ		P0G		CVD		K5Y	
N	g	max T	Nruns	mean	max	mean	max	mean	max	mean	max
8	1.7	300	5	0.05	0.39	0.07	0.41	0.06	0.38	0.01	**0.29**
10	1.7	300	5	0.04	0.36	0.08	0.36	0.06	0.36	0.01	0.24
12	1.7	300	5	0.13	0.40	0.13	0.41	0.14	0.41	0.03	0.22
15	1.7	300	5	0.05	0.35	0.08	0.40	0.09	0.34	0.02	0.21
20	1.7	300	3	0.05	0.29	0.06	0.31	0.06	0.30	0.02	0.16
15	0.0	300	5	0.01	0.04	0.02	0.04	-0.01	0.00	0.00	0.01
15	R.O.	300	5	0.00	0.05	0.02	0.06	-0.00	0.03	-0.00	0.01
15	0.8	300	5	-0.00	0.07	0.03	0.14	0.00	0.11	0.00	0.01
15	1.0	300	5	0.05	0.28	0.07	0.29	0.05	0.32	0.00	0.08
15	1.2	300	5	0.10	0.32	0.12	0.37	0.12	0.37	-0.00	0.19
15	1.5	300	5	**0.17**	0.39	0.17	0.41	0.15	**0.45**	0.03	0.20
15	2.0	300	5	0.08	0.35	0.09	0.39	0.08	0.35	0.02	0.20
15	2.5	300	5	0.02	0.30	0.04	0.30	0.04	0.27	0.00	0.18
15	3.0	300	5	0.03	0.35	0.05	0.30	0.05	0.33	0.01	0.16
15	1.5	200	7	0.16	0.33	**0.18**	0.38	**0.16**	0.38	0.00	0.14
15	1.5	250	7	**0.17**	0.38	0.17	0.43	**0.16**	0.36	0.02	0.27
12	1.5	250	28	0.13	**0.44**	0.13	0.45	0.14	0.39	0.02	0.23
12	1.7	250	26	0.14	0.41	0.14	0.41	0.15	0.44	**0.04**	0.23
15	1.5	250	28	0.16	0.43	0.16	0.45	**0.16**	0.42	0.01	0.22
15	1.7	250	24	0.13	0.38	0.15	**0.49**	0.13	0.38	0.02	0.20
20	1.5	250	27	0.14	0.37	0.15	0.45	0.13	0.38	0.01	0.24
20	1.7	250	24	0.13	0.39	0.14	0.42	0.14	0.39	0.01	0.20

Table S2: Performance of each run in finding the matching the electrostatics for each known ligand. All values shown are electrostatic overlaps (O_E); higher values are better, with 1 indicating perfect overlap. “Mean” and “max” columns show the mean and maximum values, respectively, over the set of FT frames. In each column, the entry from the best performing set is shown in bold. The special case of the “repulsive only” FT-FT electrostatic interactions is marked with “R.O” under the g column.
